# EST analysis of the scaly green flagellate *Mesostigma viride *(Streptophyta): Implications for the evolution of green plants (Viridiplantae)

**DOI:** 10.1186/1471-2229-6-2

**Published:** 2006-02-13

**Authors:** Andreas Simon, Gernot Glöckner, Marius Felder, Michael Melkonian, Burkhard Becker

**Affiliations:** 1 Botanical Institute, University of Cologne, Gyrhofstr. 15, 50931 Cologne, Germany; 2 Genome Analysis, Leibniz Institute for Age Research – Fritz Lipmann Institute, Beutenbergstr. 11, 07745 Jena, Germany

## Abstract

**Background:**

The Viridiplantae (land plants and green algae) consist of two monophyletic lineages, the Chlorophyta and the Streptophyta. The Streptophyta include all embryophytes and a small but diverse group of freshwater algae traditionally known as the Charophyceae (e.g. Charales, *Coleochaete *and the Zygnematales). The only flagellate currently included in the Streptophyta is *Mesostigma viride *Lauterborn. To gain insight into the genome evolution in streptophytes, we have sequenced 10,395 ESTs from *Mesostigma *representing 3,300 independent contigs and compared the ESTs of *Mesostigma *with available plant genomes (*Arabidopsis, Oryza*, *Chlamydomonas*), with ESTs from the bryophyte *Physcomitrella*, the genome of the rhodophyte *Cyanidioschyzon*, the ESTs from the rhodophyte *Porphyra*, and the genome of the diatom *Thalassiosira*.

**Results:**

The number of expressed genes shared by *Mesostigma *with the embryophytes (90.3 % of the expressed genes showing similarity to known proteins) is higher than with *Chlamydomonas *(76.1 %). In general, cytosolic metabolic pathways, and proteins involved in vesicular transport, transcription, regulation, DNA-structure and replication, cell cycle control, and RNA-metabolism are more conserved between *Mesostigma *and the embryophytes than between *Mesostigma *and *Chlamydomonas*. However, plastidic and mitochondrial metabolic pathways, cytoskeletal proteins and proteins involved in protein folding are more conserved between *Mesostigma *and *Chlamydomonas *than between *Mesostigma *and the embryophytes.

**Conclusion:**

Our EST-analysis of *Mesostigma *supports the notion that this organism should be a suitable unicellular model for the last flagellate common ancestor of the streptophytes. *Mesostigma *shares more genes with the embryophytes than with the chlorophyte *Chlamydomonas reinhardtii*, although both organisms are flagellate unicells. Thus, it seems likely that several major physiological changes (e.g. in the regulation of photosynthesis and photorespiration) took place early during the evolution of streptophytes, i.e. before the transition to land.

## Background

The Viridiplantae (literally meaning green plants) include all green algae and embryophyte plants. They represent a monophyletic group of organisms, which display a surprising diversity with respect to their morphology, cell architecture, life histories and reproduction, and their biochemistry. The colonization of the terrestrial habitat by streptophyte algae 450 – 470 million years ago [reviewed in [1]] was undoubtedly one of the most important steps in the evolution of life on earth [2-4], which paved the way for the evolution of the various groups of land plants (embryophytes = bryophytes, pteridophytes and spermatophytes) resulting in our current terrestrial ecosystems [5].

A thorough understanding of the evolution of land plants requires knowledge about the phylogeny of green algae and embryophytes as well as insight into the evolution of plant genomes with special reference to developmental processes. Whereas our knowledge about the phylogeny of the Viridiplantae has greatly increased over the last years, the latter has hardly been addressed to date.

The Viridiplantae are grouped into two divisions: the Chlorophyta and the Streptophyta [6]. The Chlorophyta comprise the vast majority of green algae including most scaly green flagellates (e.g. *Pyramimonas*, *Tetraselmis*), the Ulvophyceae (e.g. *Ulva*, *Acetabularia*), Chlorophyceae (e.g. *Chlamydomonas*, *Volvox*) and Trebouxiophyceae (e.g. *Chlorella*) [7,8]. The Streptophyta include all embryophyte plants and a diverse paraphyletic assemblage of freshwater green algae, the Charales (stoneworts), *Coleochaete*, the Zygnematophyceae and a few other taxa [9]. Currently, the Charales are thought to be the sister group of the embryophytes suggesting that the evolution of true land plants already started with a complex organism [10]. Remarkably, only a single scaly green flagellate *Mesostigma viride *Lauterborn, has been found to belong to the Streptophyta [10-13]. The exact phylogenetic position of *Mesostigma viride*, however, is still controversial [10-12,14-16]. *Mesostigma *has recently attracted much attention as a putative key organism for the understanding of the early evolution of the Streptophyta [17-20].

Two aspects in the evolution of land plants seem to be important in this respect. First, many key evolutionary inventions of plants took already place within the streptophyte algae. According to Graham et al. [21] one can distinguish several major transitions in the evolution of land plants starting with a *Mesostigma*-like flagellate ancestor: development of a cellulosic cell wall, multicellularity, cytokinesis by a phragmoplast, plasmodesmata, apical meristematic cell and apical cell proliferation leading to branching, asymmetric cell division, cell differentiation, retention of zygotes, heteromorphic life history, and a root meristem. Of these distinguishing features only the latter two evolved not until the embryophytes emerged. Second, the colonization of the terrestrial habitat with its exposure to air, increased solar radiation and life in a desiccating environment led to adaptations of cell architecture, metabolism and body plan to survive in the terrestrial ecosystems [5]. The evolutionary history of these adaptations is currently not known. Important questions are: How did the green algal progenitor adapt to the terrestrial habitat? Which genomic changes were associated with this transition? And which of these genes are derived from streptophyte green algae? To gain insight into these questions we have started to analyze ESTs from various streptophyte algal lineages.

Here, we present an analysis of 10,395 ESTs representing 3306 non-redundant expressed genes obtained from *Mesostigma viride*. We show that the number of genes shared is higher between *Mesostigma *and the embryophytes than between *Mesostigma *and *Chlamydomonas*. Comparison of expressed genes from *Mesostigma *with the genomes of *Arabidopsis, Chlamydomonas*, the red alga *Cyanidioschyzon*, and rice as well as ESTs from *Physcomitrella *and *Porphyra *allowed us to identify conserved and derived cellular functions within the different evolutionary lines and to obtain a first insight into the metabolic capabilities of the flagellate ancestor of green plants.

## Results

### Preparation and characterization of libraries

Total RNA was isolated from an axenic culture of *Mesostigma viride *during the light phase. The culture contained about 5 % cell division stages. The isolated RNA was used for the construction of 4 different cDNA libraries (Meso 1 – Meso 4). Meso 1 and 2 differed in the size of the cloned inserts. For Meso 3 and 4 full-length enriched cDNA was prepared and normalized prior to cloning. Meso 3 was obtained from the total normalized full-length enriched cDNA, whereas for Meso 4 the normalized full-length enriched cDNA was size-fractionated by gel permeation chromatography to remove small fragments. The basic characteristics of the four libraries are given in Table 1.

Initially, about 100–500 ESTs were sequenced from all libraries and analyzed by BLASTX against the Swissprot and translated Genbank databases. Since the Meso 2 and 4 libraries containing the larger inserts gave more promising results, we subsequently sequenced about 4000 additional ESTs from the Meso 2 and Meso 4 libraries, respectively yielding a total of 10,395 reads (5,527,413 bp). Based on comparison with published sequences from *Mesostigma viride *the rate of sequencing error was determined to be generally between 1% and 7 % (average 4 %) depending on the quality of the sequence.

ESTs were assembled using the PHRAP software yielding 3300 contigs with an average size of 769 bases (57 – 4452 bases) after manual curation. Further analysis based on sequence similarity searches revealed that 294 of these contigs were of plastidic, mitochondrial, or possibly bacterial origin (sequences showing the highest similarity to organellar or bacterial genomes, Table 2). These contigs were excluded from the data set. 1315 of the 3006 contigs analyzed (44%) showed significant similarity at the protein level to sequences from the public databases (Table 2). Hence, approximately 56% of the contigs represent either novel sequences with unknown function or untranslated regions of a gene. However, when the 1691 contigs with no significant similarity to known proteins were searched against the Interpro protein motif database, 574 (33.9%) of these contigs contained a recognizable protein motif (Table 2). The most common protein motifs found in all 3006 expressed gene sequences were bipartite nuclear localization signals (IPR001472, 197x), proline-rich regions (IPR000694, 150x) and cytochrome c heme-binding sites (IPR000345, 99x).

A functional catalogue was assembled using the 3006 *Mesostigma *contigs and the KOG-database and is presented in Table 3. As expected for an interphase cell, genes in the categories (1) translation, ribosomal structure and biogenesis (168), (2) posttranslational modification, protein turnover, chaperones (101), and (3) energy production and conversion (87) are represented by the largest number of contigs (Table 3). In the following, the assembled contigs are referred to as (expressed) genes.

### Classification of *Mesostigma *ESTs according to homologous genes in other organisms

**Figure 1:**
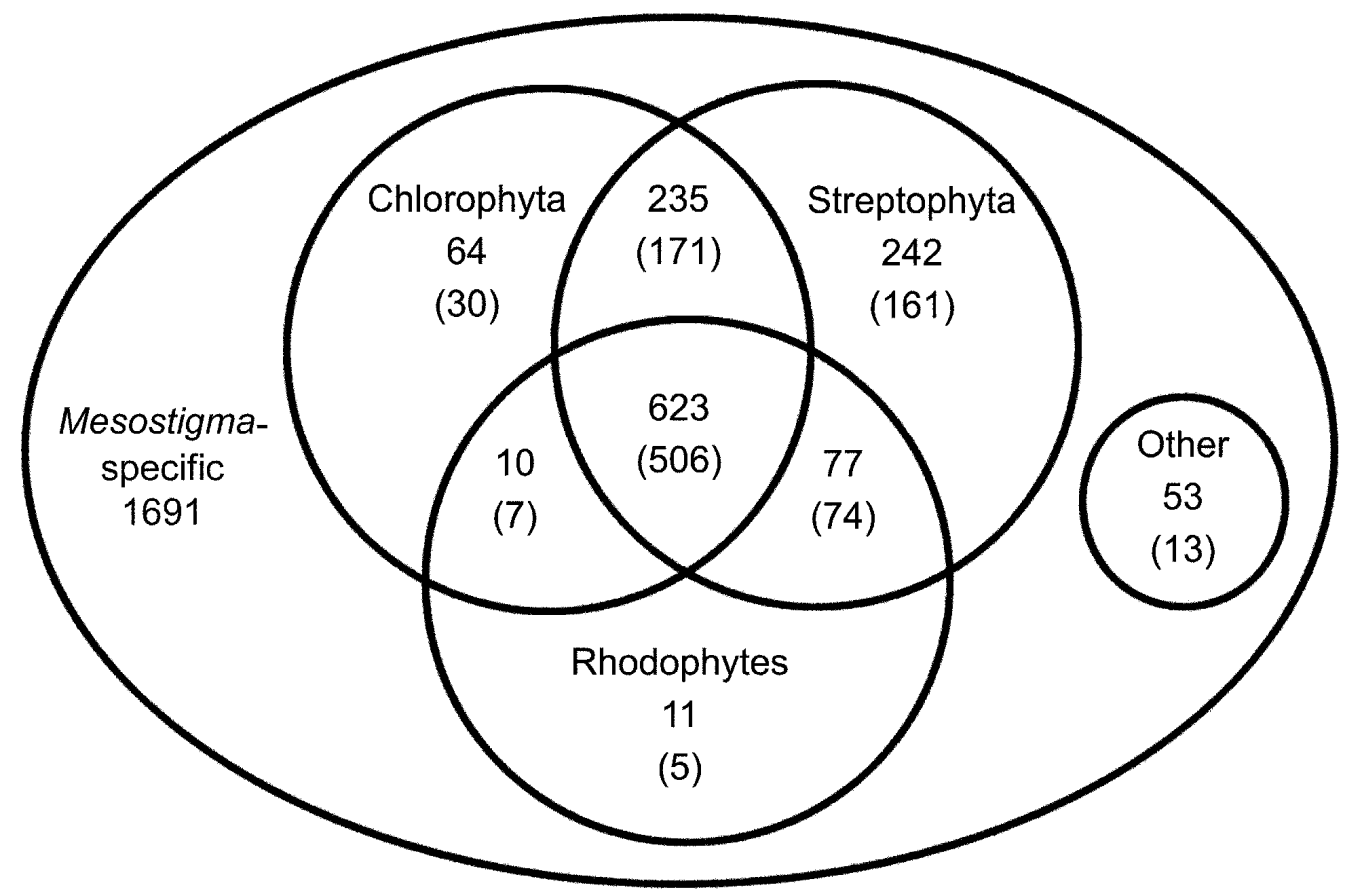
**Classification of expressed genes from *Mesostigma *according to the presence of similar proteins in other organisms in a Venn diagram**. All non-redundant expressed genes were used as a query in (t)blastx similarity searches with the Swissprot, Genbank, *Chlamydomonas*, *Cyanidioschyzon*, *Porphyra*, *Physcomitrella*, *Arabidopsis *and *Oryza *data sets. The outermost circle represents all *Mesostigma *expressed genes. The inner circles, which are labeled chlorophyte, streptophyte and rhodophyte, represent genes, which have similarity to chlorophyte, streptophyte or rhodophyte sequences, respectively. The areas depicted are not proportional to the gene numbers and the number of *Mesostigma *expressed genes in each category is written in each segment. Numbers in brackets indicate the number of expressed genes in a category after removal of low similarity hits (see Table 2 for a definition of low similarity hits).

EST data represent only a fraction of all genes of an organism. Thus, comparisons of EST data alone cannot be used to describe unique or shared genes of an organism. For embryophytes, chlorophytes and red algae complete genome sequences of at least one organism exist. This makes it possible to find potential orthologous genes if present. Moreover, the surplus of genes of an organism in respect to a complete genome can be detected in EST data. In tBLASTX analyses of the 1315 expressed genes with similarity to known proteins 90.3 % matched proteins from streptophytes, 76.1 % from chlorophytes and 61 % from rhodophytes, respectively. In addition, 46 genes showed similarity to known proteins, which have not been reported from plants or red algae to date. The overlap of *Mesostigma *genes with different organisms can be visualized in a Venn diagram (Figure 1). For 211 genes, we detected similar proteins only within the streptophyte but not in the chlorophyte or rhodophyte lineages. Conversely, for 62 genes we detected similar proteins only within the chlorophyte but not in the streptophyte or rhodophyte lineages. Surprisingly, we also found 6 genes which showed significant similarity to rhodophyte proteins but for which we could not detect any similar protein sequences within the Viridiplantae. Removal of BLAST hits with significant but low similarity (see Table 2) reduced the overall numbers to 972 expressed genes, but gave similar results (Figure 1). A complete list of genes showing only similarity to proteins with known functions present in specific subgroups of organisms can be found in supplemental Table 1 [see Additional file 1]. We will discuss important differences below.

### Overall protein similarities between various photoautotrophic organisms

To compare the overall similarity between *Mesostigma *and various photoautotrophic organisms with completed genomes or large data sets of ESTs, we decided to calculate the average identity of a protein between *Mesostigma *and the various organisms. To compare *Mesostigma *genes with the genomes or ESTs from different organisms, we calculated the average identity (AI) between *Mesostigma *and another organism as the mean value of all pair wise identities of the BLAST-matches for each organism (Table 4).

The AI between *Mesostigma *and *Chlamydomonas *or the embryophytes are very similar. The highest AI value obtained was for *Physcomitrella/Mesostigma *followed by *Arabidopsis/Mesostigma*, *Chlamydomonas/ Mesostigma *and *Oryza/Mesostigma*. The full data set includes many proteins, which we detected only in some species using *Mesostigma *expressed genes as a query. Therefore, we constructed a constrained data set (314 expressed genes, including at least 46 nuclear encoded plastidic, 9 nuclear encoded mitochondrial, and 73 cytosolic ribosomal proteins), containing only *Mesostigma *genes which gave matches with all completed genomes from photoautotrophic eukaryotic organisms (including the diatom *Thalassiosira*). This constrained data set represents a conserved core set of nuclear encoded expressed proteins from photoautotrophic eukaryote organisms. We calculated AI values for the constrained data set using complete genomes and the available ESTs of *Physcomitrella*, *Porphyra*, and *Chlamydomonas*. The results are included in Table 4. We obtained the highest AI-values in the constrained data set for the three embryophytes, followed by *Chlamydomonas*. The similar AI values for the three different embryophytes suggest that the overall evolutionary rate was very similar for the embryophytes investigated, when compared with *Mesostigma *(see below).

**Figure 2:**
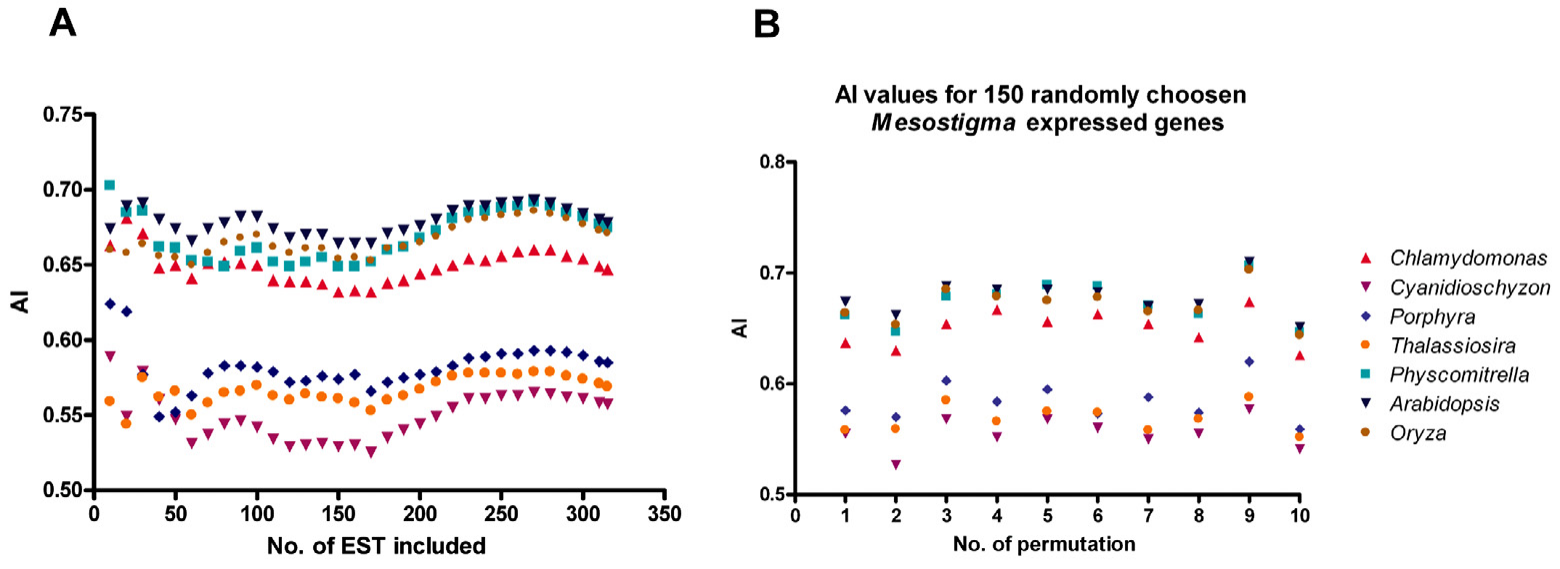
**Consistency of the constrained data set used to calculate AI values. **(A) The figure illustrates the effect of the number of genes included in the AI-values. The significant differences in the AI values are stable when more than 150 genes are included. (B) 150 genes were resampled randomly and the AIs calculated for the indicated organisms (1 – 8). AI values were calculated for the 150 most strongly (9, as revealed by the number of ESTs in a contig) and weakly (10, only single ESTs) expressed genes.

To test whether the observed differences are significant a paired students t-test was performed, and the results are shown in Table 5. Applying a significance level of 0.0072 [0.05/7 Bonferroni adjustment [22]] the differences in AI between *Mesostigma*/*Chlamydomonas *and *Mesostigma*/embryophytes are highly significant (Table 5), whereas the differences in AI among the embryophytes are not significant (Table 5). Furthermore, when we varied the numbers of expressed genes used for the calculation of the AI, we observed that when more than 100 ESTs were included the significance of the differences became very stable (Fig. 2A). In addition, to evaluate the consistency of the data set we calculated 8 times the AI for 150 randomly selected expressed genes from the constrained data set. A clear difference between the AI from the various organisms was always observed (Fig. 2B 1 – 8). The expression level of the expressed genes (as revealed by the number ESTs in a contig) had no effect on the differences between the investigated organisms (Fig. 2B, compare 9 and 10), although highly expressed genes are better conserved (Fig. 2B, 9 and 10).

Two other results are remarkable. First, for the calculation of the AI it is possible to use large EST-data sets instead of genomes. We obtained the same result for *Mesostigma*/*Chlamydomonas *genome and for *Mesostigma*/*Chlamydomonas *ESTs (AI = 0.653 for both data sets; p = 0,975, Table 5, using 244 expressed genes from *Mesostigma*). Similarly, when *Mesostigma*/*Physcomitrella *ESTs were compared with the *Mesostigma*/*Arabidopsis *genome and with the *Mesostigma*/*Oryza *genome only small differences were observed (AI = 0,675/0,681; 0,675/0,673 respectively, using 302 expressed genes from *Mesostigma*, Table 5). Statistical analysis (paired students t-test) showed that the observed differences are not significant. Furthermore, we note that the genome of the diatom *Thalassiosira pseudonana *shows a similar AI in respect to *Mesostigma *as the red algal genome and ESTs (Table 4). The difference values of these distantly related genomes represent presumably an upper threshold for reasonable AI value calculations.

### Analysis of metabolic pathways

ESTs have been widely used for the identification of metabolic pathways [23]. A complete list of all metabolic pathways identified is presented in supplemental Table 2 [see Additional file 2]. Indeed, many ESTs showed similarity to proteins required for photosynthesis (66 expressed genes), nucleotide synthesis (6), nucleotide sugar conversion, the biosynthesis of precursors of scale polysaccharides (6), heme and chlorophyll biosynthesis (6), fatty acid and lipid biosynthesis (9), terpenoid biosynthesis (6), glycolysis (11) and the TCA-cycle including pyruvate dehydrogenase and respiration (12). The biosynthetic pathways for several amino acids were also well represented in our ESTs (21 expressed genes for Ala, Arg, Gly, Ile, Leu, Lys, Pro, Ser, Thr, Trp and Val). However, for several other amino acids (Asn, Asp, Cys, Gln, Glu, His, Met, Phe, Tyr) we did not find a single EST which could be matched to the known biosynthetic pathways.

**Figure 3:**
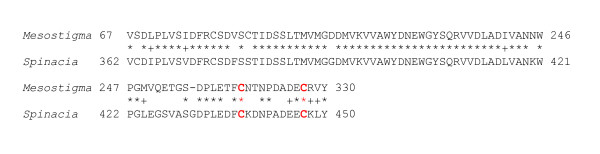
**Alignment of the deduced amino acid sequence of the putative GAPDHB (Meso2a42g12) gene from *Mesostigma *with spinach (P12860) GAPDHB**. The conserved cysteine residues are indicated in red letters. Numbers refer to the amino acid position (spinach) or nucleotide position (*Mesostigma*).

All enzymes except one (triose isomerase) of the Calvin cycle are represented by at least one EST. Interestingly, we found several genes coding for subunits of the plastidic GAPDH. In angiosperms the plastidic GAPDH consists of an A_2_B_2 _heterotetramer [24]. Compared to GAPDH A, which is present in the plastids of all eukaryotic algae, GAPDH B has a C-terminal extension that contains the two conserved cysteine residues, which are required for regulation by the thioredoxin system. To our knowledge, GAPDH B has only been reported from streptophytes. Two genes of *Mesostigma *showed significant similarity to GAPDH B from angiosperms. We present an alignment of the C-terminus of *Mesostigma *GAPDH B with the C-terminus of spinach GAPDH B in Figure 3. The two sequences are very similar and the two cysteines required for regulation by the thioredoxin system are conserved in *Mesostigma *indicating that the activity of plastidic GAPDH came under the control of the thioredoxin system early during the evolution of streptophytes. We found no evidence for a GAPDH B in *Chlamydomonas *or other chlorophytes. Therefore, the evolution of a GAPDH B might represent a molecular characteristic (synapomorphy) of the streptophytes.

A total of 25 expressed genes encode components of the light-harvesting complex. There are some light-harvesting complex proteins, which *Mesostigma *shares only with the chlorophytes and red algae (e.g. so called fucoxanthin/chlorophyll a-binding proteins). For others, we detected similar proteins only within embryophytes. However, the lhc proteins form a large superfamily and their phylogenetic analysis is beyond the scope of this study.

Several genes encode proteins of the photorespiratory C2-cycle (glycolate phosphatase, peroxisomal glycolate oxidase, a component of the glycine decarboxylase enzyme complex, and a peroxisomal serine-glyoxylate transaminase). As in embryophytes, the NADH required for reduction of hydroxy pyruvate is produced by a peroxisomal NADH malate dehydrogenase.

**Figure 4:**
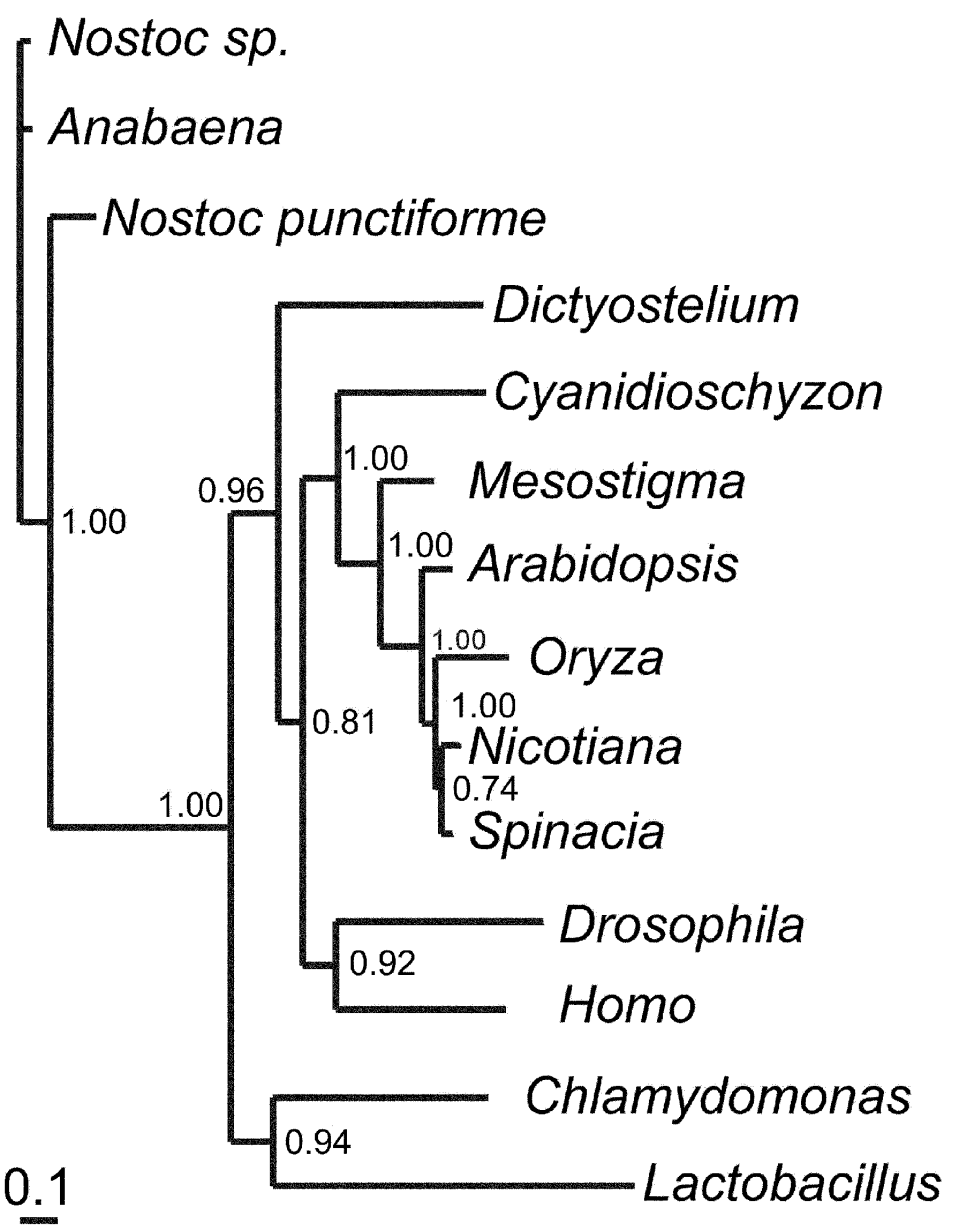
**Phylogenetic tree of glycolate oxidase and glycolate oxidase-like genes**. The tree shown was derived by Bayesian inference analysis from 402 amino acid positions using a mixed model for amino acid substitutions and a gamma correction for rate variation among sites. The Bayesian inference utilized MRBAYES, Ver. 3.0 * with posterior probabilities derived from 100000 generations and discarding a burnin of 1000. The tree obtained with a parsimony analysis using PHYLIP gave essentially the same topology.

A glycolate oxidase activity was never detected in chlorophytes by biochemical enzyme assays, but one *Chlamydomonas *protein is currently annotated as a glycolate oxidase (gene model C_340068, JGI Chlamydomonas reinhardtii v2.0) We therefore performed a phylogenetic analysis for glycolate oxidases and lactate dehydrogenases, which are both members of the same protein superfamily, from embryophytes, *Mesostigma*, *Chlamydomonas*, *Cyanidioschyzon*, *Dictyostelium*, a few metazoans and some bacteria (Fig. 4). The glycolate oxidases from embryophytes, *Mesostigma *and *Cyanidioschyzon *are monophyletic. In contrast, the glycolate oxidase-like sequence from *Chlamydomonas *clusters with bacterial sequences, which are annotated as lactate dehydrogenase and glycolate oxidases. Therefore, we conclude that, in agreements with the biochemical findings, *Chlamydomonas *does not contain a plant-type peroxisomal glycolate oxidase.

We did not find evidence for a hexokinase and sucrose biosynthesis in interphase cells of *Mesostigma*. Several ESTs represent plastidic pyruvate kinase, however, only a single EST coded for the cytosolic isoform. Expressed genes for PEP carboxylase and a cytosolic malate dehydrogenase are present, suggesting that malate may be the major substrate for respiration in the mitochondrion of *Mesostigma *as in many embryophytes. The plastidic pyruvate kinase probably functions in the generation of acetyl-CoA required to sustain fatty acid synthesis in plastids.

Scales consist mainly of the 2-keto sugar acids 3-deoxy-manno-octulosonic acid (2-keto-3-deoxy-oktonate, kdo), 5OMekdo, 3-deoxy-lyxo-heptulosaric acid, dha) and gal, galA, gul and some minor monosaccharides [25]. Expressed genes coding for kdo synthesis, and activation of kdo as CMP-kdo are present. The obtained sequence similar to a CMP-sialA transporter might actually be the CMP-kdo transporter necessary for uptake of CMP-kdo into the Golgi apparatus, as kdo and sialA are structural analogs. Interestingly, kdo-synthase and CMP-kdo-transferase are among the most conserved proteins between *Mesostigma *and the embryophytes. As in embryophytes [26], galA is synthesized via the UDP-glc dehydrogenase pathway and the myo-inositol oxygenase pathway. We could not detect the latter enzyme in *Chlamydomonas *or red algae.

Our EST-data support the presence of vitamin B12-biosynthesis and the production of a phosphagen phosphoarginine by arginine kinase in *Mesostigma*.

### Evolution of metabolism and cell structure

259 expressed genes from *Mesostigma *showed similarity to proteins belonging to various metabolic pathways. A pair-wise comparison of these genes with the genome of *Chlamydomonas *and the genomes and ESTs of the three embryophytes showed that *Mesostigma *shares more metabolic genes with the embryophytes than with *Chlamydomonas*, however, the overall AI is slightly higher with *Chlamydomonas *than with any embryophyte (AI, Table 6). Statistical analyses showed that the differences in AIs for the total metabolic enzyme data set are not significant (not shown). However, if we calculate AIs for different functional categories separately, we see that metabolic enzymes of the chloroplasts and mitochondria (photosynthesis except the Calvin cycle enzymes, fatty acid synthesis, synthesis of some amino acids, citric acid cycle, and respiration) were generally more conserved between *Mesostigma *and *Chlamydomonas *than between *Mesostigma *and the embryophytes (Table 6). In contrast, proteins of cytosolic pathways (nucleotide metabolism, NDP-sugar metabolism, and glycolysis) in *Mesostigma *were more similar to embryophyte proteins (Table 6),

Genes coding for information storage and processing, and cellular processes and signaling (Table 3) were overall more conserved between *Mesostigma *and the embryophytes than between *Mesostigma *and *Chlamydomonas*. Exceptions to this rule are proteins of the cytoskeleton (Table 7) and proteins involved in protein folding (chaperones, Table 7) and plastidic proteases (not shown), which show higher AI values with *Chlamydomonas *than with the embryophytes. If the cytoskeletal proteins are removed from the data set, the differences between *Mesostigma*/*Chlamydomonas *genome and *Mesostigma*/embryophytes are statistical significant (p = 0.000109 for *Mesostigma*/*Chlamydomonas *versus *Mesostigma*/*Physcomitrella*; p = 0.000703 for *Mesostigma*/*Chlamydomonas *versus *Mesostigma*/*Arabidopsis*, p = 0.006937 for *Mesostigma*/*Chlamydomonas *versus *Mesostigma*/*Oryza*). Remarkably, the three embryophytes behave differently in our analysis. We obtained higher AI values with *Physcomitrella *regarding the categories protein folding (chaperones), vesicular transport, transcription, and regulation (Table 7). In contrast, proteins related to DNA structure, replication, cell cycle and RNA-metabolism were more conserved between *Mesostigma *and the angiosperms *Arabidopsis *and *Oryza *than between *Mesostigma *and *Physcomitrella *(Table 7).

## Discussion

In this study, we have analyzed about 3000 expressed genes from the scaly green flagellate *Mesostigma viride*. We compared the expressed genes with the complete genomes from the angiosperms *Arabidopsis thaliana *and *Oryza sativa*, the chlorophyte *Chlamydomonas reinhardtii*, the red alga *Cyanidioschyzon merolae *and the diatom *Thalassiosira pseudonana*, as well as the ESTs from the moss *Physcomitrella patens*, and the red alga *Porphyra yezoensis*. Altogether, the *Mesostigma *proteome is more similar to the embryophytes than to *Chlamydomonas*, although *Mesostigma *and *Chlamydomonas *are both flagellate unicells. *Mesostigma *shares more genes with the embryophytes than with *Chlamydomonas*, including several enzymes confined to the streptophytes (e.g. GAPDH B, [Cu-Zn] superoxide dismutase), and the average identity of shared proteins is higher between *Mesostigma *and the embryophytes than between *Mesostigma *and *Chlamydomonas*. Therefore, we consider *Mesostigma *to be a member of the streptophytes, although *Mesostigma *clearly shares some ancestral characters with chlorophytes. Plastidic (with the exception of the Calvin cycle) and mitochondrial functions e.g. seem to be more conserved between *Mesostigma *and chlorophytes than between *Mesostigma *and embryophytes, i.e. these functions are more derived in embryophytes, probably due to adaptation of embryophytes to the terrestrial habitat. In contrast, other cellular functions except for the cytoskeleton are more conserved between *Mesostigma *and embryophytes than between *Mesostigma *and *Chlamydomonas*. Interestingly, in previous phylogenetic analyses plastidic and mitochondrial genes failed to show a clear relationship between *Mesostigma *and the streptophytes [14,15], whereas actin and nuclear-encoded SSU rDNA phylogenies support the notion that *Mesostigma *is a member of the streptophytes [10-12]. The different evolutionary rates for different cellular functions observed in this study might explain this discrepancy.

We calculated the average identity (AI) values from automatically generated BLAST output alignments. Automatically derived alignments are prone to errors. However, we believe that our approach is justified for the following reasons: (1) the BLAST alignments cover only the conserved parts of proteins and our calculated AI values indicate that in most alignments more than half of the amino acids are identical enhancing the quality of the automatically produced alignments; (2) although small mistakes may occur, they are insignificant given the high number of amino acids used to calculate the AI. On average the BLAST alignments contained about 150 amino acids and therefore about 45,000 amino acid positions were used in the constrained data set. In large data sets small unbiased errors become irrelevant [27]. Our results indicate that at least 100 (better are 150–200) expressed genes have to be used to obtain statistically significant results. It could be argued that our analysis uses only similarity values and no real evolutionary distances. AI values can be easily converted into evolutionary distances using an approximation given by Kimura [28], with the effect that the differences between the various organisms become larger but no changes occur in the order of relatedness (included in Table 4). We conclude that the AI of proteins shared between different organisms represents a reasonable measure of evolutionary relatedness, if sufficiently large data sets are used.

In the following, we briefly discuss some major differences in coding potential observed between the different photosynthetic eukaryotic organisms.

11 of 18 proteins included in supplemental Table 1 [see Additional file 1] which are shared only by *Mesostigma *and *Chlamydomonas *are associated with flagellar functions such as axonemal dyneins or components of the IFT (intra-flagellar transport) machinery. Most likely, the angiosperms lost these proteins during evolution together with the ability to produce flagellate cells. The absence of these proteins in the ESTs from the moss *Physcomitrella*, is presumably due to the fact that ESTs from developing spermatozoids are not available.

Proteins shared by *Mesostigma *and the embryophytes but not present in chlorophytes perform diverse functions. There are some well known biochemical differences between chlorophytes and streptophytes such as the presence of (Cu-Zn) superoxide dismutase [29,30] and glycolate oxidase in streptophytes [31,32] but not in chlorophytes. In addition, streptophytes use the DXP and mevalonate pathways for isoprene biosynthesis whereas chlorophytes posses only the DXP pathway [33]. For all these functions, we find molecular support in our expressed gene data set except for the mevalonate pathway of isoprene biosynthesis. Two genes matched two different enzymes of the DXP pathway; however, no matches for the MVA pathway were obtained, although the presence of this pathway has been demonstrated biochemically [33]. This could be due to the selective expression of one or the other pathway under different environmental conditions.

Remarkably, our list of proteins uniquely shared by *Mesostigma *and the embryophytes includes several proteins involved in steroid biosynthesis (e.g. a 3-oxo-5-beta-steroid dehydrogenase and a C-4 sterol oxidase), a homeobox protein of the knox family and proteins of the F-box family. The latter protein family underwent a dramatic expansion in the embryophytes (*Arabidopsis *has more than 700 members of this family).

Our expressed protein data set contains sequences similar to a protein involved in vitamin B-12 metabolism (present in rhodophytes and chlorophytes), an arginine kinase and a ARL6 protein, the latter two are absent in chlorophytes, embryophytes and red algae. It has been shown that arginine kinase is part of the ATP regeneration system in cilia of *Paramecium *[34]. *Chlamydomonas *lacks arginine kinase and recently Pazour et al. [35] showed that enzymes of the late glycolytic pathway are present in the flagella of *Chlamydomonas*, suggesting that the ATP required for flagellar function is produced by the glycolytic pathway in *Chlamydomonas*. The ARL6 protein has been implicated in protein translocation at the rER [36], although its exact function is still not known.

There are some typical embryophyte pathways that we failed to detect in *Mesostigma*, e.g. sucrose metabolism, hexokinase, and enzymes of cellulose biosynthesis. There are no reports about the presence of sucrose metabolism and hexokinase in green algae in the literature, whereas embryophyte-like Ces genes (catalytical subunit of cellulose synthase) have been reported in the streptophyte alga *Mesotaenium *[37]. Although we cannot exclude that *Mesostigma *lost these genes, we do expect to find theses genes in the genome of *Mesostigma*.

### Evolution of photosynthesis and photorespiration

It is well known that embryophytes and chlorophytes differ in important aspects of photosynthesis and its regulation, and in photorespiration (e.g., presence of GAPDHB, number of enzymes regulated by thioredoxin, glycolate oxidase vs. glycolate dehydrogenase, and presence or absence of (Cu-Zn) superoxide dismutase).

Table 8 summarizes the available information on the regulation of plastidic proteins by the thioredoxin system. The number of thioredoxin-regulated proteins has apparently increased during evolution and *Mesostigma *in this respect most closely resembles the embryophytes. Similarly, the peroxisomes of *Mesostigma *have been biochemically characterized as "leaf-type peroxisomes" [38] in full agreement with our EST-data. In contrast, chlorophytes lack glycolate oxidase and photorespiration involves only chloroplast and mitochondrial enzymes [38]. Interestingly, red algae possess a peroxisomal glycolate oxidase whereas the other enzymes of the photorespiratory cycle are located in the mitochondrion [32]. Thus, it seems likely that at the onset of streptophyte evolution major changes occurred in the regulation of the Calvin cycle and the subcellular organization of photorespiration. What might have been the driving force for these changes? We note that rhodophytes and chlorophytes both presumably evolved in a marine environment [red algae in a coastal benthic habitat, whereas chlorophytes proliferated as marine phytoplankton [39]]. Streptophyte algae most likely originated in a freshwater/brackish environment. In contrast to their marine counterparts, they had to deal with much higher light intensities and fluctuating environmental conditions such as salinity and temperature. With higher temperature, the rate of photorespiration increases. The observed changes in regulation of the Calvin cycle and photorespiration might be adaptations to this stress. It is possible that these adaptations to a shallow freshwater/brackish environment prepared streptophytes to colonize the terrestrial habitat later during evolution. In this respect we note that in extant chlorophytes activation of carbon concentrating mechanisms (CCM) is the dominant reaction to compensate for increased photorespiratory losses [38]. In contrast, streptophytes are able to channel large amounts of glycolate through the photorespiratory cycle [38]. According to Badger and Price [40] CCMs did not evolve until 400 million years ago, long after streptophytes had evolved and the colonization of the terrestrial habitat by streptophyte algae took place. Therefore during the palaeozoic era with reduced CO_2_- and increased O_2_-levels [40] streptophyte algae might have had an advantage over chlorophyte algae allowing them to colonize the terrestrial habitat during that time.

## Conclusion

In summary, our EST analysis shows that *Mesostigma *shares more genes with the embryophytes than with the chlorophyte *Chlamydomonas reinhardtii*, although both organisms are flagellate unicells. Thus, it seems likely that many typical biochemical characteristics of streptophytes evolved early during the evolution of streptophytes, i.e. before the transition to land. Alternatively, such characteristics may haven been lost in the chlorophyte lineage or remain to be discovered in other chlorophytes. A decision between these alternatives requires further information on the genomes of other preferentially early branching chlorophytes such as *Pyramimonas*.

Our EST-analysis of *Mesostigma *supports the notion that this organism should be a suitable unicellular model for the last flagellate common ancestor of the streptophytes.

## Methods

### Plant material, RNA preparation and construction of libraries

**Total RNA **was isolated from cultures of *Mesostigma viride *Lauterborn (strain NIES 476, Tsukuba, Japan) and mRNA isolated using the mTRAP™ Total Kit (Active Motif). 5 μg of mRNA were converted into cDNA using the SuperScript™ Plasmid System (Invitrogen) and the cDNA obtained was fractionated by column chromatography. A large and a small size fraction were cloned into the pSPORT1 vector (Invitrogen).

Normalized full-length cDNA was prepared by Evrogen JSC (Moscow, Russia). cDNA was prepared from total RNA using the SMART approach [41] normalized using the DSN normalization method [42] and then amplified by PCR. cDNAs were either directly cloned into a pPCR-Script Amp SK(+) Vector (PCR-Script Amp Cloning Kit, Stratagene) or a large size fraction was isolated by column chromatography and then cloned into a pGEM-T Easy vector (Promega). All libraries were transformed into TOP 10 *E. coli *cells (Invitrogen) by electroporation.

### Sequencing, contig assembly and data analysis

#### Clone preparation and sequencing

Isolated plasmids were sequenced by the cycle sequencing method using an ABI3700 96 capillary sequencer. A minimal contig set was assembled using the phrap assembler and all contigs were manually curated.

#### Annotation

Each contig was compared as 3-frame translations to the protein databases Swissprot and genpept using blastx. Furthermore, all contigs were compared using the tBLASTX search algorithm to the genome sequence of *Chlamydomonas reinhardtii*, *Cyanidioschyzon merolae*, *Thalassiosira pseudonana*, *Arabidopsis thaliana*, *Oryza sativa *and to the EST databases of *Physcomitrella patens *and *Porphyra yezoensis*. The results were compiled to an Excel compatible file. Analyses of COG and KOG categories [43,44] and Interpro protein domains [45] for the contigs were also performed.

#### Analysis of metabolic pathways

Using the metabolic pathways present at the AraCyc website [46], we identified all expressed genes with significant similarity to *Arabidopsis *genes present in AraCyc. Expressed genes that showed no significant similarity to *Arabidopsis *genes but to enzymes from other organisms were assigned to a pathway using the MetaCyc database [47].

#### Phylogenetic analysis of glycolate oxidase

Thirteen glycolate oxidase/lactate dehydrogenase sequences were obtained from public databases (*Cyanidioschyzon merolae *[KEGG:CMQ436C]; *Chlamydomonas reinhardtii *[JGI:C_340068]; *Spinacia oleracea *[Swiss-Prot:P05414]; *Nostoc punctiforme *PCC 73102 [Genbank:ZP_00106740.1]; *Nostoc *sp. PCC 7120 [Genbank:BAB77694.1]; *Anabaena variabilis *ATCC 29413 [Genbank:ZP_00160276.2]; *Arabidopsis thaliana *[Genbank:CAB78838], *Oryza sativa *[Genbank:AAB82143], *Nicotiana tabacum *[Genbank:AAC33509], *Homo sapiens *[Genbank:CAC34364], *Drosophila melanogaster *[Genbank:AAO41411], *Dictyostelium discoideum *[Genbank:XP_629946], *Lactobacillus johnsonii *NCC 533 [Genbank:NP_965805]). The nearly complete *Mesostigma *glycolate oxidase sequence was obtained by complete sequencing of EST clone Meso2b12b08. The sequences were aligned using Clustal X. The alignment was checked manually. Phylogenetic analyses were performed using the Phylip (neighbour joining and parsimony method) and MRBAYES software v 3.0 (Bayesian inference).

#### Data deposition

Sequence data from this article have been deposited with the EMBL/Genbank data libraries under accession numbers DN254242 to DN264595.

## Authors' contributions

AS constructed the libraries and participated in the analysis of the EST-data. GG sequenced the ESTs, participated in the analysis of the EST-data and helped to draft the manuscript. MF participated in the analysis of the EST-data. MM participated in the design of the study, the analysis of the EST-data and helped to draft the manuscript. BB conceived the study, and participated in its design, coordination and analysis of the EST-data, and helped to draft the manuscript. All authors read and approved the final manuscript.

## Supplementary Material

Additional file 1 Supplemental Table 1 Click here for file

Additional file 2 Supplemental Table 2 Click here for file

## Figures and Tables

**Table 1:** *Mesostigma viride *cDNA libraries used.

Name	cDNA	Number of primary clones	Percentage recombinant clones	size of inserts^1 ^(bp)	average size of inserts^1 ^(bp)	number of ESTs sequenced
Meso1	small size fraction	310 000	90	250–2000	706	100
Meso2	large size fraction	295 000	88	600–3200	1142	4954
Meso3	total cDNA normalized	106 000	63	100–1100	650	535
Meso4	large size fraction normalized	304 000	56	400–6600	2025	2403 cDNAs = 4806 ESTs^2^

^1 ^determined by agarose gel electrophoresis, ^2^sequenced from 3' and 5'end

**Table 2:** Summary of *Mesostigma viride *expressed genes obtained from four cDNA libraries (Meso 1 – Meso 4).

Category		No of contigs	
Mitochondrial^1^		36	
Plastidic^1^		65	
Bacterial^2^		193	
Novel		1691	
	with recognizable protein motif		574
	no protein motifs		1117
Similarity		1315	
	with known function^3^		574
	unknown function^4^		395
	low similarity^5^		346
Total Contigs		3300	

^1 ^sequences showing only similarity to organelle genomes. ^2 ^sequences showing only similarity to bacterial sequences or the highest similarity to bacterial sequences; the origin of these putative bacterial contaminations is currently not clear, as bacteria-free cultures of *Mesostigma *were used. ^3^similarity to proteins with a well-defined function (BLAST score >100). ^4^similarity to conserved proteins with no established function (BLAST score >100). ^5^low similarity to proteins from a few organisms (BLAST score generally between 100 and 200); might reflect conserved protein domains.

**Table 3:** Functional classification of 3006 *Mesostigma viride *contigs using the KOG system [43] and an expectation threshold of e = 10^-7^.

Functional Category	No. of Contigs
INFORMATION STORAGE AND PROCESSING	236
[J] Translation, ribosomal structure and biogenesis	168
[A] RNA processing and modification	28
[K] Transcription	26
[L] Replication, recombination and repair	9
[B] Chromatin structure and dynamics	5
	
CELLULAR PROCESSES AND SIGNALING	209
[D] Cell cycle control, cell division, chromosome partitioning	7
[Y] Nuclear structure	0
[V] Defense mechanisms	2
[T] Signal transduction mechanisms	31
[M] Cell wall/membrane/envelope biogenesis	9
[N + Z] Cytoskeleton and cell motility	18
[W] Extracellular structures	0
[U] Intracellular trafficking, secretion, and vesicular transport	41
[O] Posttranslational modification, protein turnover, chaperones	101
	
METABOLISM	212
[C] Energy production and conversion	87
[G] Carbohydrate transport and metabolism	35
[E] Amino acid transport and metabolism	24
[F] Nucleotide transport and metabolism	9
[H] Coenzyme transport and metabolism	13
[I] Lipid transport and metabolism	21
[P] Inorganic ion transport and metabolism	16
[Q] Secondary metabolites biosynthesis, transport and catabolism	7
	
POORLY CHARACTERIZED	158
[R] General function prediction only	55
[S] Function unknown	37
[X] Unnamed protein	66
Unknown	2191

**Table 4:** Comparison of the *Mesostigma *expressed genes with the genomes and ESTs from various organisms. Average identity (AI) of pair wise comparisons of *Mesostigma *expressed genes with the indicated organismal data set.

Data set (No. of contigs)	*Chlamydomonas*		*Cyanidioschyzon*	*Porphyra*	*Thalassiosira*	*Physcomitrella*	*Arabidopsis*	*Oryza*
	Genome	EST	Genome	EST	Genome	EST	Genome	Genome
All (969)	0.573	0.563	0.522	0.528	0.528	0.590	0.577	0.565
Constrained (314)	0.648	0.653 (n = 244)^1)^	0.557	0.585 (n = 188)^1)^	0.569	0.675 (n = 301)^1)^	0.679	0.671
Evolutionary distance D^2)^	0.473	0.463	0.658	0.597	0.631	0.425	0.418	0.432

The total data set contains all *Mesostigma *expressed genes with significant similarity to proteins from other organisms with known or unknown function (see Table 2). The constrained data set contains only *Mesostigma *expressed genes with significant similarity to proteins in all completely sequenced eukaryotic autotroph organisms.

^1) ^Number of ESTs showing similarity to *Mesostigma *expressed genes from the constrained data set in a tBLASTX analysis. ^2) ^Evolutionary distances were calculated using the constrained data set and the approximation given by Kimura [28]: D = -ln (1 - p - 0.2 p^2^), where p is the fraction of amino acid that differs between the two species.

**Table 5:** Statistical significance of the obtained AI values. A paired students t-test was performed for the constrained data set to test whether the observed differences between the average identity of pair wise comparisons of *Mesostigma *expressed genes with the indicated organismal data set are significant. Differences are considered significant when p is < 0.0071 (0.05/8 Bonferroni adjustment [22]).

	Variable^1^	**No. of genes shared**^**2**^	mean^3^	standard deviation^4^	t-value	Degrees of freedom	ρ
1	*Mesostigma/Chlamydomonas *G *Mesostigma/Chlamydomonas *E	244	0.652992 0.652992	0.149868 0.151255	-0,03107	243	0.975239
2	*Mesostigma/Chlamydomonas *G *Mesostigma/Physcomitrella E*	301	0.649934 0.674618	0.149292 0.153641	-3.24578	300	0.001304
3	*Mesostigma/Chlamydomonas *G *Mesostigma/Arabidopsis *G	314	0.648057 0.677994	0.148512 0.140940	-4.44025	313	0.000012
4	*Mesostigma/Chlamydomonas *G *Mesostigma./Oryza *G	314	0.648057 0.670382	0.148512 0.148384	-3.15371	313	0.001768
5	Mesostigma/Physcomitrella *E Mesostigma/Arabidopsis *G	302	0.675364 0.681159	0.153933 0.140567	-135783	301	0.175535
6	Mesostigma/Physcomitrella *E Mesostigma./Oryza *G	302	0.675364 0.673311	0.153933 0.147624	0,43158	301	0.666355
7	*Mesostigma/Arabidopsis *G *Mesostigma./Oryza *G	314	0.678730 0.671175	0.141321 0.148813	2.480053	313	0.013660

^1 ^Compared data sets E = ESTs, G = Genome. ^2 ^No. of genes shared between the compared data sets. ^3 ^AI recalculated on the basis of the genes shared between the compared datasets. ^4 ^Standard deviation of ^3^.

**Table 6:** Comparison of *Mesostigma *genes related to metabolic functions with Chlamydomonas and three embryophytes. The average identity (AI) of pair-wise comparisons of *Mesostigma *expressed genes coding for the indicated metabolic function with the ESTs or genome of the given organisms are presented.

Function^1^	*Chlamydomonas*	*Physcomitrella*	*Arabidopsis*	*Oryza*
Metabolism (259)	0.613	0.601	0.593	0.594
Plastidic metabolism (107)^2^	0,595	0,572	0,567	0,572
Mitochondrial metabolism (16)^2^	0,647	0,593	0,569	0,578
cytosolic metabolism (glycolysis, NDP-sugar metabolism, nucleotide synthesis, 22)^2^	0,568	0,639	0,641	0,632

^1 ^Numbers in brackets indicate the number of genes in this category.

**Table 7:** Comparison of *Mesostigma *genes related to cell structure functions with the genome or ESTs of Chlamydomonas and three embryophytes. The average identity of pair-wise comparisons of *Mesostigma *expressed genes coding for the indicated cellular functions with the ESTs or genomes of the given organisms are presented.

Function^1^	*Chlamydomonas*	*Physcomitrella*	*Arabidopsis*	*Oryza*
Cell structure (201)	0.629	0.641	0.627	0.618
Cytoskeleton (7)	0.751	0.729	0.0.731	0.731
Protein folding/Chaperones (21)	0.685	0.605	0.580	0.581
Cytosolic protein degradation (22)	0.682	0.733	0.722	0.703
Vesicular transport (22)	0.633	0.701	0.680	0.673
Regulation (18)	0.585	0.599	0.548	0.544
DNA structure, replication, cell cycle (21)	0.580	0.616	0.655	0.625
Transcription (16)	0.684	0.675	0.647	0.623
RNA metabolism (23)	0.599	0.619	0.637	0.637

^1 ^Numbers in brackets indicate the number of genes in this category.

**Table 8:** Regulation of plastidic enzymes by the thioredoxin system. Proteins similar to embryophyte plastidic thioredoxin-regulated proteins were identified in the genomes of *Cyanidioschyzon*, *Chlamydomonas*, and the ESTs of *Mesostigma *using the BLASTP or BLASTX algorithms. A putative thioredoxin-regulated orthologue as revealed by the conserved cysteine residues is indicated with +. An asterisk indicates putative cyanobacterial/plastidic proteins, which do not contain the conserved cysteines required for thioredoxin-regulation. Missing enzymes are indicated with -.

	**Cyanobacteria**	**Rhodophytes *Cyanidioschyzon***	**Chlorophytes *Chlamydomonas***	**Mesostigma**	**Embryophytes**
PRK	+	+	+	+	+
SDPase	*	+	+	+	+
G6PDH	*	+	+	**n.d.**	+
FBPase	*	*^1)^	+	+	+
γ-ATPase	*	*	+	+	+
GABDHB	-	-	-	+	+
NADP-MDH	-	-	+^2)^	**n.d.**	+
Rubisco activase	-^3)^	-	*	*	(+)^4)^

n.d. not detected in Mesostigma. ^1) ^In *Galdieria *(*Cyanidioschyzon*) 2 (1) of the 3 conserved cysteines occurring in the Viridiplantae are present [48]. ^2) ^Chlorophyte NADP-malate dehydrogenase possesses a C- and N-terminal extension like the embryophyte enzyme, however only the C-terminal cysteines of the embryophyte enzyme are conserved [49, 50]. ^3) ^A few cyanobacteria contain an unusual rubisco activase. Only the central AAA^+ ^domain shows similarity to plant rubisco activases, whereas the N and C terminal domain are very different [51]. ^4) ^Many angiosperms contain two forms of rubisco activase. Only the long form is regulated by the thioredoxin system [52].
