# Supplementary material for: EST analysis of the scaly green flagellate Mesostigma viride (Streptophyta): Implications for the evolution of green plants (Viridiplantae)
Source: BMC Plant Biol. 2006 Feb 13;6:2. doi: 10.1186/1471-2229-6-2 (PMC1413533; doi:10.1186/1471-2229-6-2)
Supplement: Additional file 1 — Supplemental Table 1 [file 1471-2229-6-2-S1.doc]

Supplemental Table 1: List of *Mesostigma* nuclear expressed genes showing similarity to proteins with known function which are shared only by specific subgroups of organisms. a = a protein annotated with the given function can be found in the databases, however the *Mesostigma* expressed gene showed no significant similarity to these proteins (perhaps indicating divergent proteins or protein families. LS = low similarity.

| ID no. | Length | Putative function | Green Algae | Land plants | Red algae |
| --- | --- | --- | --- | --- | --- |
| Meso2a50e06.t7 | 770 | 2-dehydro-3-deoxyphosphooctonate aldolase (EC 2.5.1.55) | G | L |  |
| Meso2a07f10.t7 | 721 | 3-beta-hydroxysteroid-delta(8),delta(7)-isomerase (EC 5.3.3.5) | G | L |  |
| Meso2a62h08.t7 | 637 | 3-deoxy-manno-octulosonate cytidylyltransferase (EC 2.7.7.38) (CMP-KDO synthetase) | G | L |  |
| Meso2a39a12.t7 | 212 | 40S ribosomal protein S10 | G | L | a |
| Meso2a12h02.t7 | 1242 | 50S ribosomal protein L35 | G | L | a |
| Meso2b15f04.r1 | 1189 | 50S ribosomal protein L9, chloroplast precursor (CL13) | G | L | a |
| Meso2a39a09.t7 | 579 | 60S ribosomal protein L26B | G | L |  |
| Meso2b22h12.r1 | 798 | Alpha-(1,4)-fucosyltransferase (EC 2.4.1.-) (FT4-M) (Galactoside 3(4)- L-fucosyltransferase) (FucTC) (AtFUT13) | G | L |  |
| Meso2b11f06.r2 | 1290 | Calcium-binding protein CaBP1 | G | L |  |
| Meso2b27h09.r1 | 1167 | Calmodulin-1/4 | G | L |  |
| Meso2a41b01.t7 | 1027 | Chlorophyll a-b binding protein 13, chloroplast precursor (LHCII type III CAB-13) | G | L |  |
| Meso2a39e12.t7 | 509 | Chlorophyll a-b binding protein 151, chloroplast precursor (LHCII type II CAB-151) (LHCP) | G | L |  |
| Meso2a49d07.t7 | 1026 | Chlorophyll a-b binding protein of LHCII type I, chloroplast precursor (CAB) (LHCP) | G | L |  |
| Meso2b22d02.t7 | 1152 | Chloroplast Drought-induced Stress Protein of 32kDa | G | L |  |
| Meso2b05f01.t7 | 963 | Chloroplast protein translocon component Tic40 precursor | G | L |  |
| Meso2a30a08.t7 | 703 | CRIPT protein | G | L |  |
| Meso2b25a01.r1 | 1095 | Cruciferin CRU1 precursor (11S globulin) (12S storage protein) | G | L |  |
| Meso2b11c08.r2 | 814 | Cysteine proteinase 2 precursor (EC 3.4.22.-) | G | L |  |
| Meso2a59b06.t7 | 644 | Dihydrodipicolinate synthase (EC 4.2.1.52) (DHDPS) | LS | LS |  |
| Meso2a46c10.t7 | 570 | Dihydropyrimidinase related protein-2 (DRP-2) (Collapsin response mediator protein 2) (CRMP-2) (N2A3) | G | L |  |
| Meso2a19c04.t7 | 646 | DNA-directed RNA polymerase III subunit 22,9 kDa polypeptide (EC 2.7.7.6) (RPC8) | G | L |  |
| Meso2a18g10.t7 | 923 | Elongation factor 3 (EF-3) | G | L |  |
| Meso2a42b09.t7 | 1907 | Ethylene responsive element binding factor 3 (AtERF3) | G | L |  |
| Meso2a56f09.t7 | 1575 | Extensin-like protein | G | L |  |
| Meso2a02.d07.t7 | 656 | F-box only protein 13 (Fragment) | G | L |  |
| Meso2a52a02.t7 | 833 | Fructose-1,6-bisphosphatase, chloroplast precursor (EC 3.1.3.11) | G | L | a |
| Meso2b14a03.r1 | 892 | Glucose-1-phosphate adenylyltransferase large subunit 1 (EC 2.7.7.27) (ADP-glucose synthase) | G | L |  |
| Meso2b08a10.t7 | 826 | Glucose-1-phosphate adenylyltransferase small subunit, chloroplast precursor (EC 2.7.7.27) (ADP-glucose synthase) | G | L |  |
| Meso2a26d10.t7 | 1269 | Hydroxyproline-rich glycoprotein | G | L |  |
| Meso2b16d07.r1 | 696 | Hypothetical 20.8 kDa protein in FGF-VUBI intergenic region (ORF 1) | G | L |  |
| Meso2a42h08.t7 | 1320 | Hypothetical 5.5 kDa protein ycf17 | G | L |  |
| Meso2b02c07.t7 | 777 | Hypothetical protein At4g14345 | G | L |  |
| Meso2b24a07.r1 | 1345 | Hypothetical protein C19A8,09 in chromosome I | G | L |  |
| Meso2b01g12.t7 | 747 | Hypothetical protein HI0020 | G | L |  |
| Meso2a46c07.t7 | 777 | Hypothetical protein pr46A | G | L |  |
| Meso2a25a06.t7 | 376 | Lactoylglutathione lyase (EC 4.4.1.5) (Methylglyoxalase) (Aldoketomutase) (Glyoxalase I) (Glx I) | G | L | a |
| Meso2b15h01.r1 | 1372 | lipoate-protein ligase A-like | G | L |  |
| Meso2b14e01.t7 | 1186 | Mitochondrial import receptor subunit TOM22 homolog (Translocase of outer membrane 22 kDa subunit homolog) | G | L |  |
| Meso2a23h08.t7 | 533 | one helix protein | G | L |  |
| Meso2a37f05.t7 | 580 | Phosphoenolpyruvate carboxylase (EC 4.1.1.31) (PEPCase) | G | L | a |
| Meso2a07c02.t7 | 507 | Photosystem I reaction center subunit psaK, chloroplast precursor (Photosystem I subunit X) (PSI-K) | G | L | a |
| Meso2a28h06.t7 | 1255 | Photosystem I reaction center subunit V, chloroplast precursor (PSI- G) | G | L |  |
| Meso2a11c08.t7 | 1424 | Photosystem II 10 kDa polypeptide, chloroplast precursor | G | L |  |
| Meso2b18h04.r1 | 700 | Plastocyanin major isoform, chloroplast precursor (DNA-damage- repair/toleration protein DRT112) | G | L |  |
| Meso2b22f01.t7 | 898 | Plenty-of-prolines-101; POP101; SH3-philo-protein | G | L |  |
| Meso2b25a03.r1 | 1372 | Probable prefoldin subunit 2 | G | L |  |
| Meso2a59a02.t7 | 450 | Protease Do-like 8, chloroplast precursor (EC 3.4.21.-) | G | L |  |
| Meso2a26b04.t7 | 676 | Protein c20orf139 | G | L |  |
| Meso2b11e09.t7 | 870 | Protein C11orf10 (HSPC005) | G | L |  |
| Meso2a51f10.t7 | 670 | Protein phosphatases PP1 regulatory subunit sds22 | G | L |  |
| Meso2a11d12.t7 | 507 | Putative cuticle collagen C09G5,5 | G | L |  |
| Meso2a64g03.t7 | 634 | putative secretory carrier membrane protein | G | L |  |
| Meso2b10c02.r1 | 1308 | Retrotransposable element Tf2 155 kDa protein type 2 | G | L |  |
| Meso2b13g02.t7 | 754 | Retrovirus-related Pol polyprotein from transposon TNT 1-94 | G | L |  |
| Meso2b17g08.t7 | 1515 | Ribulose bisphosphate carboxylase/oxygenase activase, chloroplast precursor | G | L |  |
| Meso2b25c08.r1 | 1269 | Sensor protein luxQ (EC 2.7.3.-) | G | L |  |
| Meso2b17e02.r1 | 1121 | Similar to dihydropyrimidine dehydrogenase | G | L |  |
| Meso2a62g11.t7 | 751 | SNF8 like protein | G | L |  |
| Meso2a63a11.t7 | 487 | SON protein (SON3) (Negative regulatory element-binding protein) (NRE- binding protein) (DBP-5) | G | L |  |
| Mesob01b12.t7 | 625 | Solute carrier family 2, facilitated glucose transporter, member 8 (Glucose transporter type 8) | G | L |  |
| Meso2b19c06.t7 | 1018 | Spore coat protein SP96 | G | L |  |
| Meso2b22d11.r1 | 1548 | Syntaxin 72 (AtSYP72) | G | L |  |
| Meso2b10b01.t7 | 686 | Thiol:disulfide interchange protein txlA homolog | G | L |  |
| Meso2b14d02.r1 | 741 | Transcription elongation factor B polypeptide 1 (RNA polymerase II transcription factor SIII subunit C) (SIII p15) | G | L |  |
| Meso2b04a12.r1 | 799 | U6 snRNA-associated Sm-like protein LSm7 | G | L |  |
| Meso2b14c06.t7 | 682 | ubiquitin-like protein | G | L |  |
| Meso2b18g07.r1 | 819 | Unknown thylakoid lumen protein, chloroplast precursor | G | L |  |
| Meso2b21h11.r1 | 1268 | Vacuolar protein sorting 29 (Vesicle protein sorting 29) (hVPS29) (MDS007) (PEP11) (DC7/DC15) | G | L |  |
| Meso2a29g08.t7 | 798 | Vegetative cell wall protein gp1 precursor (Hydroxyproline-rich glycoprotein 1) | G | L |  |
| Meso2a63b08.t7 | 1615 | Vegetative cell wall protein gp1 precursor (Hydroxyproline-rich glycoprotein 1) | G | L |  |
| Meso2b13h11.t7 | 822 | Vegetative cell wall protein gp1 precursor (Hydroxyproline-rich glycoprotein 1) | G | L |  |
| Meso2b11b01.r2 | 1440 | Vesicle transport v-SNARE 13 (AtVTI13) (Vesicle transport v-SNARE protein VTI13) | G | L |  |
| Meso2b08g11.r1 | 1221 | Vesicle-associated membrane protein 722 (AtVAMP722) (Synaptobrevin- related protein 1) | G | L |  |
| Meso2b20h11.t7 | 1350 | Vesicle-associated membrane protein 722 (AtVAMP722) (Synaptobrevin- related protein 1) | G | L |  |
| Meso2b13f07.t7 | 1595 | Zinc finger protein 593 (Zinc finger protein T86) | G | L |  |
| Meso2b09c09.t7 | 1486 | Zinc finger protein CONSTANS-LIKE 2 | G | L |  |
| Meso2a12e07.t7 | 1118 | Chlorophyll a-b binding protein L1818, chloroplast precursor | G |  | R |
| Meso2b26h03.t7 | 1169 | Cob(I)alamin adenosyltransferase, mitochondrial precursor (EC 2.5.1.17) (Methylmalonic aciduria type B homolog) | G |  | R |
| Meso2b09a08.r1 | 1392 | DNA-binding protein H | G |  | R |
| Meso2b18f02.r1 | 1152 | Elongation factor 2 kinase (EC 2.7.1.-) (eEF-2 kinase) (eEF-2K) (Calcium/calmodulin-dependent eukaryotic elongation factor-2 kinase) |  |  | R |
| Meso2b28f02.r1 | 1209 | Glutathione S-transferase 1 (EC 2.5.1.18) (GST class-sigma) | G |  | R |
| Meso2a20f07.t7 | 1516 | Glutathione S-transferase 1 (EC 2.5.1.18) (GST class-sigma) | G |  | R |
| Meso2a50c12.t7 | 650 | Protein KIAA1404 (Fragment) | G |  | R |
| Meso2a09c11 | 653 | Putative Mg^2+^-transporter | G |  | R |
| Meso2b04b08.r1 | 1140 | 13 kDa deflagellation-inducible protein | G |  |  |
| Meso2b06e03.t7 | 1106 | 13 kDa deflagellation-inducible protein | G |  |  |
| Meso2a03f12.t7 | 705 | cGMP-inhibited 3'.5'-cyclic phosphodiesterase B (EC 3.1.4.17) (Cyclic GMP inhibited phosphodiesterase B) | G |  |  |
| Meso2b13d10.t7 | 793 | Cytoplasmic dynein light chain (T-complex testis-specific protein 1 homolog) (Protein CW-1). | G |  |  |
| Meso2b13h04.r1 | 977 | Dynein 14 kDa light chain, flagellar outer arm | G |  |  |
| Meso2b18d08.t7 | 778 | Dynein light chain 2B, cytoplasmic | G |  |  |
| Meso2a61f02.t7 | 393 | Hydroxylamine reductase (EC 1.7.-.-) (Hybrid-cluster protein) (HCP) | G |  |  |
| Meso2b16d06.r1 | 664 | Selenoprotein W | G |  |  |
| Meso2a02.c09.t7 | 683 | Troponin C, slow skeletal and cardiac muscles (TN-C) | G |  |  |
| Meso2b12b08.t7 | 814 | Urea amidolyase [Includes: Urea carboxylase (EC 6.3.4.6); Allophanate hydrolase (EC 3.5.1.54)] | G |  |  |
| Meso2a16e01.t7 | 613 | (S)-2-hydroxy-acid oxidase, peroxisomal (EC 1.1.3.15) (Glycolate oxidase) (GOX) (Short chain alpha-hydroxy acid oxidase) | a | L | R |
| Meso2a66c08.t7 | 677 | 24-dehydrocholesterol reductase precursor (EC 1.3.1.-) (3-beta- hydroxysterol delta-24-reductase) (Seladin-1) (Diminuto/dwarf1 homolog) |  | L | R |
| Meso2b02g06.r1 | 572 | 50S ribosomal protein L21, mitochondrial precursor | 1) | L | R |
| Meso2b12g03.t7 | 1000 | Actin-depolymerizing factor 3 (ADF 3) (ZmABP3) (ZmADF3) |  | L | R |
| Meso2b10e04.r1 | 899 | Bundle sheath defective protein 2 |  | L | R |
| Meso2b11e03.r2 | 1692 | CMP-sialic acid transporter (CMP-Sia-Tr) (CMP-SA-Tr) (Solute carrier family 35 member A1) |  | L | R |
| Meso2b26c07.t7 | 854 | Covalently-linked cell wall protein 14 precursor (Inner cell wall protein) |  | L | R |
| Meso2a42d06.t7 | 771 | Cyanobacterial phytochrome B (EC 2.7.3.-) |  | L | R |
| Meso2a60d06.t7 | 481 | Cytochrome c oxidase subunit Vb precursor | a | L | R |
| Meso2a15b01.t7 | 1123 | D-3-phosphoglycerate dehydrogenase, chloroplast precursor (EC 1.1.1.95) (3-PGDH) | a | L | R |
| Meso2b28g09.t7 | 771 | F-box/LRR-repeat protein 2 (F-box and leucine-rich repeat protein 2) |  | L | R |
| Meso2a30c05.t7 | 664 | F-box/LRR-repeat protein 2-like |  | L | R |
| Meso2b02d12.t7 | 1163 | F-box/LRR-repeat protein 2-like |  |  | R |
| Meso2b10a09.r1 | 1330 | F-box/LRR-repeat protein 7 (F-box and leucine-rich repeat protein 7) |  | L | R |
| Meso2a47h03.t7 | 621 | Guanine deaminase (EC 3.5.4.3) (Guanase) (Guanine aminase) (Guanine aminohydrolase) (GAH) (GDEase) |  | L | R |
| Meso2b07e09.r1 | 765 | Hypothetical 14.6 kDa protein in QAH/OAS sulfhydrylase 3'region |  | L | R |
| Meso2b18g03.t7 | 1878 | Hypothetical protein C713,10 in chromosome II |  | L | R |
| Meso2b24a04.r1 | 1422 | Hypothetical protein RP167 |  | L | R |
| Meso2b11a05.r2 | 1091 | Hypothetical protein sll1388 |  | L | R |
| Meso2b05h02.t7 | 1359 | Hypothetical protein ywdK |  | L | R |
| Meso2b10c03.r1 | 608 | MCT-1 protein-like |  | L | R |
| Meso2b02d04.r1 | 1263 | Mitochondrial import inner membrane translocase subunit Tim9 |  | L | R |
| Meso2b27h12.r1 | 869 | Na(+)-translocating NADH-quinone reductase subunit F (EC 1.6.5.-) (Na(+)-translocating NQR subunit F) (Na(+)-NQR subunit F) |  | L | R |
| Meso2b11e05.r2 | 1163 | NADH dehydrogenase I (Complex I) alpha subcomplex 1 (MWFE) | a | L | R |
| Meso2a24h09.t7 | 592 | NADH dehydrogenase-like protein |  | L | R |
| Meso2a43d07.t7 | 764 | Oxygen-evolving enhancer protein 3-1, chloroplast precursor (OEE3) (16 kDa subunit of oxygen evolving system of photosystem II) | a | L | R |
| Meso2a25a11.t7 | 710 | Probable diphthine synthase (EC 2.1.1.98) (Diphthamide biosynthesis methyltransferase) | a | L | R |
| Meso2b08c07.r1 | 1085 | Putative 60S ribosomal protein YPL183BW, mitochondrial precursor |  | L | R |
| Meso2b25a10.r1 | 1050 | Putative 60S ribosomal protein YPL183BW, mitochondrial precursor |  | L | R |
| Meso2a06b08.t7 | 742 | Putative dioxygenase |  | L | R |
| Meso2b18a10.r1 | 1145 | Putative fiber protein Fb14 |  | L | R |
| Meso2a23g02.t7 | 470 | Putative fatty acid hydroxylase | a | L | R |
| Meso2a27g04.t7 | 658 | Ubiquitin-conjugating enzyme E2-18 kDa (EC 6.3.2.19) (Ubiquitin- conjugating enzyme 15) (Ubiquitin-protein ligase) |  | L | R |
| Meso2b16b11.r1 | 1372 | UXT protein (Ubiquitously expressed transcript protein) |  | L | R |
| Meso2b02d08.r1 | 1085 | Very hypothetical protein C1672,04c in chromosome III |  | L | R |
| Meso2b23e03.t7 | 1091 | 28 kDa heat- and acid-stable phosphoprotein (PDGF-associated protein) |  | L |  |
| Meso2a13d01.t7 | 451 | 3-oxo-5-beta-steroid 4-dehydrogenase (EC 1.3.99.6) (Delta(4)-3- ketosteroid 5-beta-reductase) (Aldo-keto reductase family 1 member D1) |  | L |  |
| Meso2b02h04.r1 | 1303 | 50S ribosomal protein L18 |  | L |  |
| Meso2a28f08.t7 | 883 | 50S ribosomal protein L7/L12 | a | L | a |
| Meso2a06b06.t7 | 775 | 60S ribosomal protein L28 | a | L |  |
| Meso2b11c06.t7 | 583 | 66 kDa stress protein (p66) | a | L | a |
| Meso2a23a06.t7 | 443 | Actin A |  | L |  |
| Meso2b11b03.t7 | 679 | ASC1 | a | L |  |
| Meso2a55b08.t7 | 721 | Baculoviral IAP repeat-containing protein 3 (Inhibitor of apoptosis protein 1) (HIAP1) (HIAP-1) (C-IAP2) |  | L |  |
| Mesob03b01.t7 | 597 | beta-carotene hydrolase |  | L |  |
| Meso2b09d01.t7 | 1062 | C-4 methyl sterol oxidase (EC 1.-.-.-) |  | L |  |
| Meso2b22h11.r1 | 1011 | Checkpoint serine/threonine-protein kinase BUB1 (EC 2.7.1.-) |  | L |  |
| Meso2a39b10.t7 | 806 | Chlorophyll a-b binding protein 8, chloroplast precursor (LHCII type I CAB-8) |  | L |  |
| Meso2a05b07.t7 | 1219 | Chloroplast 50S ribosomal protein L29 |  | L | a |
| Meso2b03c07.t7 | 508 | Chloroplast mRNA-binding protein CSP41 precursor | a | L |  |
| Meso2b17c10.r1 | 742 | Chloroplast nucleoid DNA-binding protein -like protein |  | L |  |
| Mesob03g12.t7 | 766 | Down syndrome critical region protein 5 homolog |  | L |  |
| Meso2b23e01.r1 | 1281 | F-box/LRR-repeat protein 2 (F-box and leucine-rich repeat protein 2) |  | L |  |
| Meso2b01e05.r1 | 865 | Ferredoxin-thioredoxin reductase, variable chain (FTR-V) (Ferredoxin- thioredoxin reductase subunit A) |  | L |  |
| Meso2a20f12.t7 | 392 | Fructose-bisphosphate aldolase, cytoplasmic isozyme (EC 4.1.2.13). | a | L |  |
| Meso2a12g10.t7 | 749 | GDP-mannose 4,6-dehydratase (EC 4.2.1.47) (GDP-D-mannose dehydratase), |  | L |  |
| Meso2a07f02.t7 | 1153 | Glyceraldehyde 3-phosphate dehydrogenase B, chloroplast precursor (EC 1.2.1.13) |  | L |  |
| Meso2a42g12.t7 | 423 | Glyceraldehyde 3-phosphate dehydrogenase B, chloroplast precursor (EC 1.2.1.13) |  | L |  |
| Meso2b14e12.r1 | 932 | Heat shock factor binding protein 1 |  | L |  |
| Meso2b06a06.r1 | 724 | Homeobox protein PKNOX1 (PBX/knotted homeobox 1) (Homeobox protein PREP-1), |  | L |  |
| Meso2b17b05.t7 | 1041 | Hydrophobic protein RCI2A (Low temperature and salt responsive protein LTI6A), | a | L |  |
| Meso2a49d01.t7 | 223 | Leucine rich repeat protein-related |  | L |  |
| Mesob05c10.t7 | 164 | Leucine-rich repeat resistance protein-like protein [Gossypium hirsutum] |  | L |  |
| Meso2b17c07.r1 | 1025 | Light regulated protein precursor |  | L |  |
| Meso2b20a04.r1 | 809 | Major pollen allergen Lig v 1 |  | L |  |
| Meso2a05d11.t7 | 720 | Mitochondrial import receptor subunit TOM7-1 (Translocase of outer membrane 7 kDa subunit 1) |  | L |  |
| Mesob04g12.t7 | 556 | Mitotic checkpoint protein-like |  | L |  |
| Meso2b13d01.r1 | 596 | Nuclear transport factor 2 (NTF-2) | a | L |  |
| Meso2b18d05.r1 | 958 | Plastid ribosomal protein S6 | a | L | a |
| Meso2a41h07.t7 | 238 | Probable glycerophosphoryl diester phosphodiesterase 2 precursor (EC 3.1.4.46) | a | L |  |
| Meso2b08a08.t7 | 1071 | Probable prefoldin subunit 4 (ABI3-interacting protein 3) | a | L |  |
| Mesob05a03.t7 | 811 | Protein C6orf115 (PRO2013) |  | L |  |
| Meso2b01h01.t7 | 1117 | Protein disulfide isomerase precursor (EC 5.3.4.1) (PDI) (Endosperm protein E-1) | a | L |  |
| Meso2b28a12.r1 | 936 | Protein transport protein SEC61 beta subunit |  | L |  |
| Meso2a28a02.t7 | 331 | Putative bifunctional nuclease |  | L |  |
| Meso2b23g05.r1 | 1245 | Putative glycosylation enzyme |  | L |  |
| Meso2a44g02.t7 | 617 | Putative prenylcysteine oxidase precursor (EC 1.8.3.5) | a | L |  |
| Meso2b01e02.t7 | 1016 | Ras-related protein RAB1BV | a | L |  |
| Meso2b02d03.t7 | 676 | Similar to peptidyl-prolyl cis-trans isomerase |  | L |  |
| Meso1a01.b01.t7 | 320 | Superoxide dismutase [Cu-Zn] (EC 1.15.1.1) |  | L |  |
| Meso2b05h09.r1 | 678 | Transcription initiation factor IIB (General transcription factor TFIIB) |  | L | a |
| Meso2b20a12.r1 | 842 | Troponin C, isoform 2B |  | L |  |
| Meso2b18d10.r1 | 710 | Ubiquinol-cytochrome C reductase complex 8,0 kDa protein (EC 1.10.2.2) |  | L |  |
| Meso2a51a07.t7 | 784 | Ubiquinol-cytochrome C reductase complex ubiquinone-binding protein QP-C (EC 1.10.2.2) | a | L |  |
| Meso2b04a05.t7 | 998 | Violaxanthin de-epoxidase | a | L |  |
| Meso2a26d07.t7 | 1039 | Wound-induced basic protein |  | L |  |
| Meso2a62c01.t7 | 1039 | Glutathione S-transferase (EC 2.5.1.18) |  |  | R |
| Meso2b09h09.r1 | 1184 | Hypothetical 14.3 kDa protein A-211C6,1 |  |  | R |
| Meso2b12b11.t7 | 818 | Ribonuclease III (EC 3.1.26.3) (RNase III) |  |  | R |
| Meso2a01.h04.t7 | 383 | ADP-ribosylation factor-like protein 6 |  |  |  |
| Meso2a22g07.t7 | 312 | Arginine kinase (EC 2.7.3.3) (AK) |  |  |  |
| Meso2b21g03.r1 | 1475 | Dynein light chain 4, axonemal |  |  |  |
| Meso2b08h04.t7 | 1382 | Glutathione S-transferase (EC 2.5.1.18) | a | a | a |
| Meso2a02.a04.t7 | 613 | Hypothetical protein yfdE |  |  |  |
| Mesob02g12.t7 | 1352 | Retinoblastoma-binding protein 9 (RBBP-9) (B5T overexpressed gene protein) (Bog protein) |  |  |  |
| Meso2b06b08.t7 | 721 | Sperm surface protein Sp17 (Sperm autoantigenic protein 17) |  |  |  |
| Meso2a14g05.t7 | 661 | Thioredoxin (TRX) | a | a | a |

^1)^ only one protein for plastids and mitochondria in *Chlamydomonas*? (dual targeting)?
