# Supplementary material for: EST analysis of the scaly green flagellate Mesostigma viride (Streptophyta): Implications for the evolution of green plants (Viridiplantae)
Source: BMC Plant Biol. 2006 Feb 13;6:2. doi: 10.1186/1471-2229-6-2 (PMC1413533; doi:10.1186/1471-2229-6-2)
Supplement: Additional file 2 — Supplemental Table 2 [file 1471-2229-6-2-S2.doc]

Supplemental Table 2: Metabolic pathways in *Mesostigma viride*. Expressed genes from *Mesostigma* showing similarity (threshold e-value < e-10) to known *Arabidopsis* pathways (Aracyc). In cases that the expressed genes from *Mesostigma* yielded more than one protein hit in the *Arabidopsis* AraCyc database, only the one is presented for which a higher e-value was obtained.

Aerobic glycerol catabolism

AT1g42970 Meso2a42g12.t7 (7.3e-34) Glyceraldehyde 3-phosphate dehydrogenase B

AT1g74030 Meso2a47g10.t7 (7.3e-66) Enolase

AT1g79530 Meso2a07f02.t7 (2.0e-86) Glyceraldehyde 3-phosphate dehydrogenase B

Meso2a25h09.t7 (3.6e-128) Glyceraldehyde 3-phosphate dehydrogenase

AT3g26650 Meso2a40a08.t7 (2.5e-127) Glyceraldehyde 3-phosphate dehydrogenase A

Meso2a62e05.t7 (3.3e-34) Glyceraldehyde 3-phosphate dehydrogenase A

AT3g55440 Meso2b04a03.r1 (8.0e-76) Triosephosphate isomerase, cytosolic

Meso2b04a03.t7 (1.2e-35) Triosephosphate isomerase, cytosolic

AT5g08570 Meso2a13g10.t7 (2.0e-38) Pyruvate kinase, cytosolic isozyme

AT5g52920 Meso2a24h04.t7 (5.7e-50) Pyruvate kinase 1

AT3g12780 Meso2a05f08.t7 (2.8e-144) Phosphoglycerate kinase, chloroplast precursor

Alanine biosynthesis II

AT1g70580 Meso2b02g08.r1 (2.2e-57) Alanine aminotransferase 2

Alanine degradation

AT1g70580 Meso2b02g08.r1 (2.2e-57) Alanine aminotransferase 2

Arabinose catabolism

AT3g01850 Meso2b17f07.t7 (1.3e-34) Ribulose-phosphate 3-epimerase, chloroplast precursor

Arginine biosynthesis 4

AT4g24830 Meso2a44b08.t7 (7.1e-91) Argininosuccinate synthase, chloroplast precursor

Arginine biosynthesis II

AT4g24830 Meso2a44b08.t7 (7.1e-91) Argininosuccinate synthase, chloroplast precursor

Ascorbate biosynthesis

AT2g45790 Meso2b19h03.r1 (4.5e-82) Phosphomannomutase

Biosynthesis of chlorophyll

AT1g03630 Meso2a18f10.t7 (8.0e-28) Protochlorophyllide reductase C, chloroplast precursor

AT2g40490 Meso2b08c04.r1 (1.7e-32) Uroporphyrinogen decarboxylase

Meso2b08d04.t7 (6.0e-13) Uroporphyrinogen decarboxylase

AT4g27440 Meso2a54d07.t7 (2.0e-102) Protochlorophyllide reductase, chloroplast precursor

AT5g63570 Meso2a48c07.t7 (4.8e-62) Glutamate-1-semialdehyde 2,1-aminomutase

Biosynthesis of proto- and siroheme

AT2g40490 Meso2b08c04.r1 (1.7e-32) Uroporphyrinogen decarboxylase

Meso2b08d04.t7 (6.0e-13) Uroporphyrinogen decarboxylase

AT5g63570 Meso2a48c07.t7 (4.8e-62) Glutamate-1-semialdehyde 2,1-aminomutase

Biotin biosynthesis I

AT2g43360 Meso2a18c05.t7 (1.4e-112) Biotin synthase

AT5g57590 Meso2a15h12.t7 (1.8e-37) Adenosylmethionine-8-amino-7-oxononanoate aminotransferase

Brassinosteroid biosynthesis

AT3g19820 Meso2a66c08.t7 (4.7e-39) 24-dehydrocholesterol reductase precursor

Calvin cycle

AT1g32060 Meso2b11a11.t7 (2.0e-86) Phosphoribulokinase, chloroplast precursor

AT2g01140 Meso2a58d02.t7 (1.1e-133) Fructose-bisphosphate aldolase, chloroplast precursor

AT2g45290 Meso2b07h01.r1 (1.0e-10) Transketolase, chloroplast precursor

AT3g01850 Meso2b17f07.t7 (1.3e-34) Ribulose-phosphate 3-epimerase, chloroplast precursor

AT3g12780 Meso2a05f08.t7 (2.8e-144) Phosphoglycerate kinase, chloroplast precursor

AT3g55440 Meso2b04a03.r1 (8.0e-76) Triosephosphate isomerase, cytosolic

Meso2b04a03.t7 (1.2e-35) Triosephosphate isomerase, cytosolic

AT3g55800 Meso2a44f07.t7 (9.4e-121) Sedoheptulose-1,7-bisphosphatase, chloroplast precursor

AT4g26520 Meso2a48e10.t7 (1.0e-84) Fructose-bisphosphate aldolase, cytoplasmic isozyme

AT5g38410 Meso2a13d03.t7 (1.5e-40) Ribulose bisphosphate carboxylase small chain, chloroplast precursor

Meso2a20d01.t7 (2.0e-40) Ribulose bisphosphate carboxylase small chain, chloroplast precursor

AT1g42970 Meso2a42g12.t7 (7.3e-34) Glyceraldehyde 3-phosphate dehydrogenase B

AT1g79530 Meso2a07f02.t7 (2.0e-86) Glyceraldehyde 3-phosphate dehydrogenase B

Meso2a25h09.t7 (3.6e-128) Glyceraldehyde 3-phosphate dehydrogenase

AT3g26650 Meso2a40a08.t7 (2.5e-127) Glyceraldehyde 3-phosphate dehydrogenase A

Meso2a62e05.t7 (3.3e-34) Glyceraldehyde 3-phosphate dehydrogenase A

At3g54050 Meso2a52a02.t7 Fructose-1,6-bisphosphatase, chloroplast precursor

Carotenoid biosynthesis

AT1g08550 Meso2b04a05.t7 (1.2e-17) violaxanthin de-epoxidase

AT4g14210 Meso2a32g03.t7 (7.9e-37) Phytoene dehydrogenase

AT5g67030 Meso2a42f06.t7 (1.8e-20) Zeaxanthin epoxidase, chloroplast precursor

Meso2a63h12.t7 (6.6e-14) zeaxanthin epoxidase precursor

Chorismate biosynthesis

AT4g39980 Meso2a35h12.t7 (2.6e-27) Phospho-2-dehydro-3-deoxyheptonate aldolase 1, chloroplast precursor

Deoxypyrimidine nucleotide/side metabolism

AT3g46940 Meso2b09e12.t7 (9.0e-13) Deoxyuridine 5'-triphosphate nucleotidohydrolase

AT4g09320 Meso2a01.d03.t7 (1.9e-58) Nucleoside diphosphate kinase B

AT5g28050 Meso2a47h03.t7 (3.4e-36) Guanine deaminase

AT5g59440 Meso2b15g01.r1 (4.0e-58) ???

(Deoxy)ribose phosphate metabolism

AT2g45290 Meso2b07h01.r1 (1.0e-10) Transketolase, chloroplast precursor

AT5g28050 Meso2a47h03.t7 (3.4e-36) Guanine deaminase

Ethylene biosynthesis from methionine

AT1g02500 Meso2a19f11.t7 (5.2e-175) S-adenosylmethionine synthetase 2

Fatty acid biosynthesis, initial steps

AT1g36050 Meso2b08g07.r1 (2.5e-19) ???

AT5g46290 Meso2b20e03.t7 (2.3e-108) 3-oxoacyl-[acyl-carrier-protein] synthase I, chloroplast precursor

Fatty acid elongation, saturated

AT2g22230 Meso2a28c05.t7 (3.4e-34)

AT5g46290 Meso2b20e03.t7 (2.3e-108) 3-oxoacyl-[acyl-carrier-protein] synthase I, chloroplast precursor

At1g24360 Meso2b21a10.r1 3-oxoacyl-[acyl-carrier protein] reductase, chloroplast precursor

Fatty acid elongation, unsaturated

AT2g22230 Meso2a28c05.t7 (3.4e-34) 3-keto-acyl-ACP dehydratase

AT5g46290 Meso2b20e03.t7 (2.3e-108) 3-oxoacyl-[acyl-carrier-protein] synthase I, chloroplast precursor

FormylTHF biosynthesis

AT4g13890 Meso2a50c11.t7 (1.1e-39) Serine hydroxymethyltransferase, mitochondrial precursor

AT4g32520 Meso2a48a08.t7 (9.2e-59) Serine hydroxymethyltransferase, mitochondrial precursor

Galactose metabolism

AT4g20460 Meso2a24d10.t7 (2.2e-50) UDP-glucose 4-epimerase

Gluconeogenesis

AT1g42970 Meso2a07f02.t7 (2.0e-86) Glyceraldehyde 3-phosphate dehydrogenase B

Meso2a42g12.t7 (7.3e-34) Glyceraldehyde 3-phosphate dehydrogenase B

AT1g56190 Meso2a05f08.t7 (3.6e-144) Phosphoglycerate kinase, chloroplast precursor

AT1g74030 Meso2a47g10.t7 (7.3e-66) Enolase

AT1g79530 Meso2a25h09.t7 (3.6e-128) Glyceraldehyde 3-phosphate dehydrogenase

AT2g01140 Meso2a58d02.t7 (1.1e-133) Fructose-bisphosphate aldolase, chloroplast precursor

AT3g12780 Meso2a05f08.t7 (2.8e-144) Phosphoglycerate kinase, chloroplast precursor

AT3g26650 Meso2a40a08.t7 (2.5e-127) Glyceraldehyde 3-phosphate dehydrogenase A

Meso2a62e05.t7 (3.3e-34) Glyceraldehyde 3-phosphate dehydrogenase A

AT4g26530 Meso2a48e10.t7 (1.0e-84) Fructose-bisphosphate aldolase, cytoplasmic isozyme

AT3g55440 Meso2b04a03.r1 (8.0e-76) Triosephosphate isomerase, cytosolic

Meso2b04a03.t7 (1.2e-35) Triosephosphate isomerase, cytosolic

Glyceraldehyde 3-phosphate catabolism

AT1g42970 Meso2a07f02.t7 (2.0e-86) Glyceraldehyde 3-phosphate dehydrogenase B

Meso2a42g12.t7 (7.3e-34) Glyceraldehyde 3-phosphate dehydrogenase B

AT1g74030 Meso2a47g10.t7 (7.3e-66) Enolase

AT1g79530 Meso2a25h09.t7 (3.6e-128) Glyceraldehyde 3-phosphate dehydrogenase

AT3g12780 Meso2a05f08.t7 (2.8e-144) Phosphoglycerate kinase, chloroplast precursor

AT3g26650 Meso2a40a08.t7 (2.5e-127) Glyceraldehyde 3-phosphate dehydrogenase A

Meso2a62e05.t7 (3.3e-34) Glyceraldehyde 3-phosphate dehydrogenase A

Glycine biosynthesis

AT1g22020 Meso2a50c11.t7 (1.1e-39) Serine hydroxymethyltransferase, mitochondrial precursor

AT4g32520 Meso2a48a08.t7 (9.2e-59) Serine hydroxymethyltransferase, mitochondrial precursor

Glycolysis

AT1g42970 Meso2a07f02.t7 (2.0e-86) Glyceraldehyde 3-phosphate dehydrogenase B

Meso2a42g12.t7 (7.3e-34) Glyceraldehyde 3-phosphate dehydrogenase B

AT1g74030 Meso2a47g10.t7 (7.3e-66) Enolase

AT1g79530 Meso2a25h09.t7 (3.6e-128) Glyceraldehyde 3-phosphate dehydrogenase

AT2g01140 Meso2a58d02.t7 (1.1e-133) Fructose-bisphosphate aldolase, chloroplast precursor

AT3g12780 Meso2a05f08.t7 (2.8e-144) Phosphoglycerate kinase, chloroplast precursor

AT3g26650 Meso2a40a08.t7 (2.5e-127) Glyceraldehyde 3-phosphate dehydrogenase A

Meso2a62e05.t7 (3.3e-34) Glyceraldehyde 3-phosphate dehydrogenase A

AT3g55440 Meso2b04a03.r1 (8.0e-76) Triosephosphate isomerase, cytosolic

Meso2b04a03.t7 (1.2e-35) Triosephosphate isomerase, cytosolic

AT4g26530 Meso2a48e10.t7 (1.0e-84) Fructose-bisphosphate aldolase, cytoplasmic isozyme

AT5g08570 Meso2a13g10.t7 (2.0e-38) Pyruvate kinase, cytosolic isozyme

AT5g52920 Meso2a24h04.t7 (5.7e-50) Pyruvate kinase 1

Glycolysis 4

AT1g42970 Meso2a07f02.t7 (2.0e-86) Glyceraldehyde 3-phosphate dehydrogenase B

Meso2a42g12.t7 (7.3e-34) Glyceraldehyde 3-phosphate dehydrogenase B

AT1g74030 Meso2a47g10.t7 (7.3e-66) Enolase

AT1g79530 Meso2a25h09.t7 (3.6e-128) Glyceraldehyde 3-phosphate dehydrogenase

AT2g01140 Meso2a58d02.t7 (1.1e-133) Fructose-bisphosphate aldolase, chloroplast precursor

AT3g12780 Meso2a05f08.t7 (2.8e-144) Phosphoglycerate kinase, chloroplast precursor

AT3g26650 Meso2a40a08.t7 (2.5e-127) Glyceraldehyde 3-phosphate dehydrogenase A

Meso2a62e05.t7 (3.3e-34) Glyceraldehyde 3-phosphate dehydrogenase A

AT3g55440 Meso2b04a03.r1 (8.0e-76) Triosephosphate isomerase, cytosolic

Meso2b04a03.t7 (1.2e-35) Triosephosphate isomerase, cytosolic

AT5g03690 Meso2a48e10.t7 (5.5e-84) Fructose-bisphosphate aldolase, cytoplasmic isozyme

AT5g08570 Meso2a13g10.t7 (2.0e-38) Pyruvate kinase, cytosolic isozyme

AT5g52920 Meso2a24h04.t7 (5.7e-50) Pyruvate kinase 1

Glycosylglyceride desaturation pathway

AT3g15850 Meso2a10d11.t7 (5.5e-68) Acyl-CoA desaturase 1

AT4g30950 Meso2b25c11.t7 (2.3e-140) Omega-6 fatty acid desaturase, chloroplast precursor

Glyoxylate cycle

AT2g44350 Meso2a26b10.t7 (3.4e-28) Citrate synthase, mitochondrial precursor

AT4g35830 Meso2a54h06.t7 (1.2e-48) Aconitate hydratase, cytoplasmic

Homogalacturonan degradation

AT1g04630 Meso2a62f11.t7 (4.1e-33) ????

Homoserine biosynthesis

AT3g02020 Meso2a24e07.t7 (3.2e-10) Aspartate kinase

Isoleucine biosynthesis I

AT3g23940 Meso2b19g07.r1 (1.8e-09) Dihydroxy-acid dehydratase

AT3g48560 Meso2a32d05.t7 (2.4e-74) Acetolactate synthase I, chloroplast precursor

Meso2a45d04.t7 (1.5e-58) Acetolactate synthase I, chloroplast precursor

AT3g58610 Meso2a10h10.t7 (2.7e-30) Ketol-acid reductoisomerase, chloroplast precursor

Meso2a43g11.t7 (4.0e-17) Ketol-acid reductoisomerase, chloroplast precursor

Meso2a52a08.t7 (1.9e-42) Ketol-acid reductoisomerase, chloroplast precursor

AT5g16290 Meso2a06c06.t7 (1.1e-39) Acetolactate synthase small subunit

Lactose degradation

AT1g30620 Meso2a24d10.t7 (5.7e-50) UDP-glucose 4-epimerase

Leucine biosynthesis

AT2g43090 Meso2a34d08.t7 (9.9e-46) 3-isopropylmalate dehydratase small subunit 1

AT4g13430 Meso2b21c03.r1 (1.1e-76) 3-isopropylmalate dehydratase large subunit 1

Leucine degradation I

AT4g34030 Meso2b03a08.r1 (1.5e-14) methylcrotonyl-CoA carboxylase

Lysine and diaminopimelate biosynthesis

AT3g02020 Meso2a24e07.t7 (3.2e-10) Aspartate kinase

AT3g14390 Meso2b11e07.r2 (1.9e-97) Diaminopimelate decarboxylase

AT3g53580 Meso2a25b08.t7 (6.8e-47) Diaminopimelate epimerase 2

Mannose and GDP-mannose metabolism

AT2g45790 Meso2b19h03.r1 (4.5e-82) Phosphomannomutase

Methionine degradation 1

AT1g02500 Meso2a19f11.t7 (5.2e-175) S-adenosylmethionine synthetase 2

AT4g13940 Meso2a35b04.t7 (6.7e-16) Adenosylhomocysteinase

Meso2a63c03.t7 (3.4e-68) Adenosylhomocysteinase

Non-oxidative branch of the pentose phosphate pathway

AT2g45290 Meso2b07h01.r1 (1.0e-10) Transketolase, chloroplast precursor

AT3g01850 Meso2b17f07.t7 (1.3e-34) Ribulose-phosphate 3-epimerase, chloroplast precursor

AT3g02360 Meso2a45f12.t7 (7.1e-64) 6-phosphogluconate dehydrogenase, decarboxylating

Non-phosphorylated glucose catabolism

AT1g74030 Meso2a47g10.t7 (7.3e-66) Enolase

AT5g08570 Meso2a13g10.t7 (2.0e-38) Pyruvate kinase, cytosolic isozyme

AT5g52920 Meso2a24h04.t7 (5.7e-50) Pyruvate kinase 1

Nucleotide metabolism

AT4g09320 Meso2a01.d03.t7 (1.9e-58) Nucleoside diphosphate kinase B

Oxidative branch of the pentose phosphate pathway

AT3g02360 Meso2a45f12.t7 (7.1e-64) 6-phosphogluconate dehydrogenase, decarboxylating

Phospholipid desaturation pathway

AT4g30950 Meso2b25c11.t7 (2.3e-140) Omega-6 fatty acid desaturase, chloroplast precursor

Photorespiration

AT4g18360 Meso2a16e01.t7 (1.6e-68) Glycolate oxidase

AT5g38410 Meso2a13d03.t7 (1.5e-40) Ribulose bisphosphate carboxylase small chain, chloroplast precursor

AT5g38430 Meso2a20d01.t7 (2.0e-40) Ribulose bisphosphate carboxylase small chain, chloroplast precursor

Purine biosynthesis

AT3g55010 Meso2a30g07.t7 (2.0e-40) Phosphoribosylformylglycinamidine cyclo-ligase, chloroplast precursor

AT4g34740 Meso2a65e08.t7 (3.4e-84) Amidophosphoribosyltransferase, chloroplast precursor

PRPP biosynthesis

AT2g45290 Meso2b07h01.r1 (1.0e-10)

Pyrimidine ribonucleotide/ribonucleoside metabolism

AT4g09320 Meso2a01.d03.t7 (1.9e-58)

AT5g28050 Meso2a47h03.t7 (3.4e-36)

Ribitol utilization

AT3g01850 Meso2b17f07.t7 (1.3e-34)

Ribose catabolism

AT2g45290 Meso2b07h01.r1 (1.0e-10)

Serine biosynthesis

AT4g34200 1 Meso2a15b01.t7 (1.3e-84)

Serine-isocitrate lyase pathway

AT1g74030 Meso2a47g10.t7 (7.3e-66)

AT2g42790 Meso2a06d11.t7 (6.1e-46)

AT2g44350 Meso2a26b10.t7 (3.4e-28)

AT4g13890 Meso2a50c11.t7 (1.1e-39)

AT4g35830 Meso2a54h06.t7 (1.2e-48)

AT4g37930 Meso2a48a08.t7 (9.2e-59)

Starch biosynthesis

AT5g19220 Meso2b14a03.r1 (8.9e-16)

AT5g48300 Meso2b08a10.t7 (2.4e-24)

Sucrose biosynthesis

AT1g43670 Meso2a44f07.t7 (1.4e-22)

Superpathway for gluconate utilization

AT3g02360 Meso2a45f12.t7 (7.1e-64)

tRNA charging pathway

AT1g29880 Meso2a45a06.t7 (8.8e-40)

AT3g02660 Meso2a07e02.t7 (3.7e-31)

AT4g17300 Meso2a41b04.t7 (1.6e-30)

TCA cycle, aerobic respiration

AT2g42790 1 Meso2a06d11.t7 (6.1e-46)

AT2g44350 1 Meso2a26b10.t7 (3.4e-28)

AT4g35830 2 Meso2a54h06.t7 (1.2e-48)

Tryptophan biosynthesis

AT4g27070 Meso2a11e05.t7 (3.7e-94)

AT5g48220 Meso2a28g05.t7 (2.0e-31)

Meso2a44e01.t7 (1.9e-12)

UDP-glucose conversion

AT1g15690 Meso2a51h11.t7 (4.2e-21)

AT2g18230 Meso2b06b02.t7 (1.2e-67)

AT4g20460 Meso2a24d10.t7 (2.2e-50)

AT5g09650 Meso2a11g03.t7 (1.9e-74)

Ureide biosynthesis

AT4g34900 Meso2a26c09.t7 (3.7e-26)

Valine biosynthesis

AT3g23940 Meso2b19g07.r1 (1.8e-09)

AT3g48560 Meso2a32d05.t7 (2.4e-74)

Meso2a45d04.t7 (1.5e-58)

AT3g58610 Meso2a10h10.t7 (2.7e-30)

Meso2a43g11.t7 (4.0e-17)

Meso2a52a08.t7 (1.9e-42)

AT5g16290 Meso2a06c06.t7 (1.1e-39)

Xanthophyll cycle

AT1g08550 Meso2b04a05.t7 (1.2e-17)

AT5g67030 Meso2a42f06.t7 (1.8e-20)

Meso2a63h12.t7 (6.6e-14)

Xylulose-monophosphate cycle

AT1g43670 Meso2a44f07.t7 (1.4e-22)

AT2g01140 Meso2a58d02.t7 (1.1e-133)

AT2g45290 Meso2b07h01.r1 (1.0e-10)

AT3g54050 Meso2a52a02.t7 (3.9e-05)

AT4g26530 Meso2a48e10.t7 (1.0e-84)
